# Functionally prioritised whole-genome sequence variants improve the accuracy of genomic prediction for heat tolerance

**DOI:** 10.1186/s12711-022-00708-8

**Published:** 2022-02-19

**Authors:** Evans K. Cheruiyot, Mekonnen Haile-Mariam, Benjamin G. Cocks, Iona M. MacLeod, Raphael Mrode, Jennie E. Pryce

**Affiliations:** 1 School of Applied Systems Biology, La Trobe University, Bundoora, VIC 3083 Australia; 2 Agriculture Victoria Research, AgriBio, Centre for AgriBiosciences, Bundoora, VIC 3083 Australia; 3 International Livestock Research Institute, Nairobi, Kenya; 4 Scotland’s Rural College, Edinburgh, UK

## Abstract

**Background:**

Heat tolerance is a trait of economic importance in the context of warm climates and the effects of global warming on livestock production, reproduction, health, and well-being. This study investigated the improvement in prediction accuracy for heat tolerance when selected sets of sequence variants from a large genome-wide association study (GWAS) were combined with a standard 50k single nucleotide polymorphism (SNP) panel used by the dairy industry.

**Methods:**

Over 40,000 dairy cattle with genotype and phenotype data were analysed. The phenotypes used to measure an individual’s heat tolerance were defined as the rate of decline in milk production traits with rising temperature and humidity. We used Holstein and Jersey cows to select sequence variants linked to heat tolerance. The prioritised sequence variants were the most significant SNPs passing a GWAS p-value threshold selected based on sliding 100-kb windows along each chromosome. We used a bull reference set to develop the genomic prediction equations, which were then validated in an independent set of Holstein, Jersey, and crossbred cows. Prediction analyses were performed using the BayesR, BayesRC, and GBLUP methods.

**Results:**

The accuracy of genomic prediction for heat tolerance improved by up to 0.07, 0.05, and 0.10 units in Holstein, Jersey, and crossbred cows, respectively, when sets of selected sequence markers from Holstein cows were added to the 50k SNP panel. However, in some scenarios, the prediction accuracy decreased unexpectedly with the largest drop of − 0.10 units for the heat tolerance fat yield trait observed in Jersey cows when 50k plus pre-selected SNPs from Holstein cows were used. Using pre-selected SNPs discovered on a combined set of Holstein and Jersey cows generally improved the accuracy, especially in the Jersey validation. In addition, combining Holstein and Jersey bulls in the reference set generally improved prediction accuracy in most scenarios compared to using only Holstein bulls as the reference set.

**Conclusions:**

Informative sequence markers can be prioritised to improve the genomic prediction of heat tolerance in different breeds. In addition to providing biological insight, these variants could also have a direct application for developing customized SNP arrays or can be used via imputation in current industry SNP panels.

**Supplementary Information:**

The online version contains supplementary material available at 10.1186/s12711-022-00708-8.

## Background

Heat tolerance is the ability of an animal to maintain its production and reproduction levels under hot and humid conditions. With increasing global warming effects on animal production, there is worldwide growing desire to breed for resilience to heat, in part, to meet the demand of the increasing human population while coping with the challenges of hot and ever-changing production environments [1]. Dairy cows are often prone to heat stress due to the elevated metabolic heat of lactation. Temperature and humidity levels exceeding the thresholds that are considered as comfortable for the dairy cows and other farm animals can compromise production (reduced milk, growth, etc.), reproduction (e.g., reduced conception rates), and welfare (increased thirst and hunger), leading to substantial economic losses [2].

Considerable research has been conducted in many countries to assess heat tolerance and performance in farm livestock, including measuring changes in core body temperatures (e.g., rectal, vaginal, rumen, etc.) and thermal indices [e.g., temperature–humidity index (THI)] [3]. To study the effect of THI on milk production of dairy cows, Ravagnolo et al. [4] introduced a method in which daily milk records are merged with temperature-humidity data to measure the rate of milk decline associated with changes in heat stress. This method has been widely adopted in many countries [5–7] due to the availability of extensive test-day milk records from dairy farms and climate data from weather stations.

In Australia, Nguyen et al. [7] used test-day milk records (milk, fat, and protein yield) and climate data collected from across Australia’s dairying regions to evaluate heat tolerance in dairy cattle, which culminated in the release to the dairy industry [through DataGene Ltd; (https://datagene.com.au/)] of the first genomic breeding values for this trait in 2017, with an average reliability of 38%. While current prediction estimates are promising, even a smaller lift in reliability is economically important to the wider industry since the genetic improvement is linearly related to the selection intensity, accuracy of estimated breeding values (EBV), genetic variation and is inversely proportional to the generation interval [8, 9]. The accuracy of prediction is the only component that is influenced by research in different ways to drive genetic improvement for a given trait whereas the other components (selection intensity, genetic variation, and generation cycle) are largely controlled by breeding companies and farmers.

Besides increasing the size of the reference population, one way to boost the accuracy of prediction is to increase the density of markers used in genomic predictions. However, replacing single nucleotide polymorphism (SNP) panels by the full set of whole-genome sequence variants has, in most cases, yielded limited, or no appreciable increase in the accuracy of prediction for various traits in cattle [10], sheep [11], and avian species [12]. Alternatively, a substantial increase in accuracy of prediction has been realized by augmenting standard industry SNP panels (e.g., a 50k SNP array) with a small set of informative or causal mutations for a trait [11, 13–15]. To fully maximize predictions, this approach requires a careful selection of informative markers. Thanks to the 1000 Bull Genomes project [16], it is now possible to use this sequence database to impute genotypes to the whole-genome sequence. This may facilitate a more accurate selection of highly informative variants for genomic predictions, especially for complex traits such as heat tolerance. Specifically, having a large sample size and high-resolution genotypes can help to identify many putative causal variants with medium- and small-sized effects.

In addition to sample size, the composition of the population used for discovering informative variants can have an impact on the genomic predictions of a trait. Several studies e.g., [17–19] have reported that the mapping precision of the causal variants underlying traits is improved in multi-breed compared to single-breed genome-wide association studies (GWAS), especially for quantitative trait loci (QTL) that segregate across breeds [19]. In a simulated study, van den Berg et al. [14] demonstrated that using variants that are close to the causal mutations can improve genomic predictions. With real data, Raymond et al. [20] found that the accuracy of prediction for stature increased when candidate variants that were discovered from a meta-GWAS of 17 cattle populations were used. In sheep, Moghaddar et al. [11] reported an enhanced accuracy of prediction for various production traits when they used pre-selected variants from the QTL discovery set that comprised multiple breed compositions. Besides these studies and several others that used single-breed sets to discover variants for traits, e.g., [15, 21], there is still a dearth of information on the value of variants that are discovered from multi-breed populations in genomic predictions. Notably, it is critical to ensure that the population(s) used to discover informative sequence variants for a trait is (are) independent of that used to train subsequent genomic predictions to avoid bias, as demonstrated by [22].

The main objective of this study was to quantify the accuracy of prediction of heat tolerance in Holsteins when sets of selected sequence markers from a GWAS based on a large sample of Holstein cows were added to the standard-industry 50k SNP panel that is routinely used for genomic evaluations in Australia. The selected variants are likely linked to causal mutations that underpin the genetic basis for heat tolerance [23] and, therefore, could enable more accurate and sustained genomic selection for heat tolerance. In addition, we investigated the accuracy of prediction when informative sequence markers discovered in Holstein cows are used in the genomic predictions of numerically smaller breeds, including Jersey and crossbred cattle. Moreover, we investigated the gain in accuracy of prediction when using informative markers discovered in a combined set of Holstein and Jersey cows (i.e., a multi-breed QTL discovery set). Finally, we compared the gain in accuracy when single-breed (Holstein bulls) versus multi-breed (Holstein + Jersey bulls) reference sets are used in the genomic predictions.

## Methods

### Phenotypes

The phenotypes were obtained from DataGene (DataGene Ltd., Melbourne, Australia; https://datagene.com.au/), and included test-day milk, fat, and protein yield for Holstein, Jersey, and Holstein–Jersey crossbred cows collected from dairy herds between 2003 and 2017 that were combined with climate data (daily temperature and humidity) obtained from weather stations across Australia’s dairying regions. The distribution of dairy herds and weather stations, data filtering, and the calculation of environmental covariate [i.e., temperature–humidity index (THI)] used in this work were described previously [23, 24].

The rate of decline (slope) in milk, fat, and protein yields due to heat stress events was estimated using reaction norm models described by [24]. Briefly, data on milk, fat, or protein yields were adjusted for the fixed effects, including herd-test-day, year-season of calving, parity, Legendre polynomials (order 3) on the cow age on the day of the test, and the Legendre polynomials (order 8) on the interaction between parity and DIM. The number of records (tests) per Holstein bull (N = 3323) ranged from 4 to 263,067 and that per Jersey bull (N = 852) ranged from 5 to 54,242. The number of daughters per Holstein bull ranged from 1 to 18,613 with an average of 149, and that per Jersey bull ranged from 1 to 3169 with an average of 88.8. The random non-genetic effect fitted in the model included a random regression on a linear orthogonal polynomial of THI, where the intercept represents the level of mean milk yield, and the linear component represents the change in milk yield (slope) due to heat stress for each cow, and a residual term. The reaction norm models [4] were used in the analyses with the THI threshold set at 60 (i.e., if THI < 60, then THI = 60) based on previous work in Australia [7, 25] showing that milk yield traits begin to decline at this THI threshold. The analyses to derive trait deviations (TD**)**, which represent phenotypes adjusted for all fixed effects (i.e., the slope for each cow) were conducted using ASReml v4.2 [26]. Slope solutions (i.e., TD) for each bull’s daughters were averaged to obtain heat tolerance slope traits for bulls and were equivalent to daughter trait deviations (DTD). As in [27], the DTD in this study should be treated as approximations equivalent to the averages of daughter phenotypes since the models did not include pedigree data. Notably, the derivation of intercept and slope traits in this initial step was necessary because of the computational resources required to fit complex models and the large sample size in our study. From here on, the slope traits derived from milk, fat, and protein yield records are referred to as heat tolerance milk (HTMYslope), fat (HTFYslope), and protein (HTPYslope).

### Genotypes

Two genotype datasets were prepared for the above cows and bulls with heat tolerance phenotypes: the standard 50k SNP chip (i.e., Illumina 50k Bovine Bead Chip used in previous work in Australia [7] and 15,098,486 imputed whole-genome sequence variants (WGS). The WGS was imputed [28] using the genomic sequence data from Run7 of the 1000 Bull Genome Project based on the ARS-UCD1.2 reference genome (http://1000bullgenomes.com/), and variants were filtered on the estimated imputation accuracy (*R*^2^ > 0.4) and minor allele frequency (MAF > 0.005). The detailed imputation procedure is described in [23].

### Study design: discovery, reference, and validation datasets

**Fig. 1:**
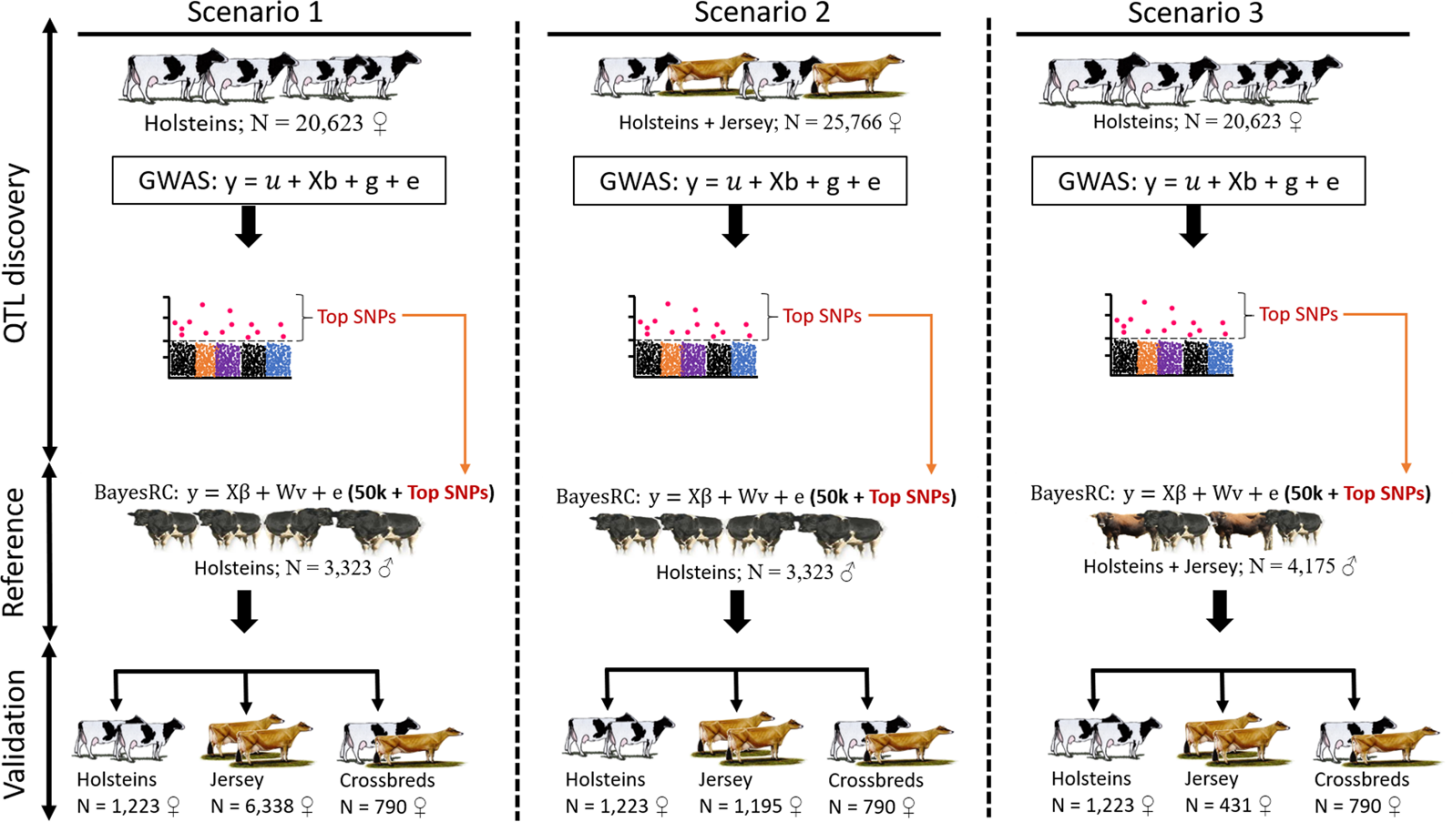
Overview of the analyses with the three study scenarios. ‘Scenario 1’: the QTL discovery set was comprised of a subset of 20,623 older Holstein cows (born in 2012 or earlier); the reference set included only Holstein bulls (N = 3323) that were not sires of cows in the discovery set; validation sets included Holsteins, Jersey, and crossbred cows. ‘Scenario 2’: the QTL discovery set was comprised of a combined set of Holsteins (N = 20,623) and Jersey cows (N = 5143); the reference set included only Holstein bulls (N = 3323; as described for “Scenario 1”) that were not sires of the Holstein cows in the discovery set; validation sets included Holstein (N = 1223), Jersey (N = 6338) and crossbred (N = 790) cows. ‘Scenario 3’: the QTL discovery set included only Holstein cows (N = 20,623; as described for “Scenario 1”); the reference set included a combined set of Holsteins (N = 3323) and Jersey (N = 852) bulls); validation sets included Holstein (N = 1223), Jersey (N = 431) and crossbred (N = 790) cows

The animals with genotypes and heat tolerance phenotypes included Holsteins (29,107 ♀/3323 ♂), Jerseys (6338 ♀/1364 ♂), and Holstein–Jersey crossbreds (790 ♀/0 ♂). These animals were split into three independent groups to achieve the specific objectives: (i) a QTL discovery set that was used to discover informative sequence markers for heat tolerance, (ii) a reference set that was used to develop genomic prediction equations, and (iii) independent validation sets that were used to assess genomic prediction accuracy. The validation sets included three breed subsets: Holstein, Jersey, and crossbred cows. Across all the prediction scenarios, we ensured that the QTL discovery set used in the GWAS was independent of the reference set used in genomic predictions to minimise bias in the predictions [22]. The different sets of animals used for each group (QTL discovery, reference, and validation) are described schematically in Fig. 1, with a more detailed description in the following paragraphs.

#### Scenario 1

Scenario 1 aimed at testing the value of pre-selected sequence variants from Holstein cows in the genomic prediction of the same breed as well as in the prediction of other numerically smaller breeds, including Jersey and crossbred cows: (i) a QTL discovery set that included 20,623 Holstein cows born in 2012 or earlier; (ii) a reference set that included 3323 Holstein bulls with none of these bulls siring the cows in the discovery set to ensure the independence of the phenotypes between the two datasets; and (iii) three validation sets, i.e. (a) that was comprised of 1223 younger Holstein cows (born in 2013 or later), which were not daughters of the Holstein bulls used in the reference set, (b) that included 6338 Jersey cows, and (c) that included 790 crossbred cows. Each of the three validation sets was randomly split into two subsets of approximately equal size (see Additional file 1: Table S1) to facilitate the calculation of standard errors of prediction.

#### Scenario 2

Scenario 2 aimed at testing whether pre-selected informative markers from a multi-breed population improves the accuracy of predictions compared to pre-selected markers from the single-breed QTL discovery set: (i) a QTL discovery set that included older cows i.e. Holstein (N = 20,623 ♀; born in 2012 or earlier) and Jersey (N = 5143 ♀; calved for the first time in 2014 or earlier); (ii) a reference set that comprised Holstein bulls (N = 3323); (iii) three validation sets, i.e. (a) Holstein cows (N = 1223 ♀; as described for “Scenario 1”), (b) Jersey cows (N = 1195; younger cows that calved for the first time in 2014 or later); and (c) crossbred cows (N = 790; as for “Scenario 1”). Each validation set was randomly split into two subsets (see Additional file 1: Table S1), and these were the same subsets used in “Scenario 1” for Holsteins and crossbreds.

#### Scenario 3

Scenario 3 aimed at testing the accuracy of prediction when using a multi-breed reference set as follows: (i) a QTL discovery set of Holstein cows (N = 20,623; born in 2012 or earlier as described for “Scenario 1”, i.e., the single-breed discovery set); (ii) a reference set that consisted of a multi-breed set of Holstein bulls (N = 3323 ♂; as for “Scenario 1”) and Jersey bulls (N = 852 ♂); and (iii) three validation sets, i.e. (a) Holstein cows (N = 1223; as for “Scenarios 1 and 2”), (b) Jersey cows (N = 431) that were not daughters of the bulls used in the multi-breed reference set; and (c) crossbred cows (N = 790; as for “Scenarios 1 and 2”). Validation sets were split into two subsets, and for Holstein and crossbred validation they were the same subsets as in “Scenarios 1 and 2”.

### QTL discovery and selection of informative markers (‘top SNPs’)

To identify informative sequence variants for heat tolerance traits (using the “discovery” sets described above), we performed a GWAS using mixed linear models to test associations between individual SNPs and cow’s slope traits using the GCTA software [29]. The details of the GWAS for the Holstein discovery set are described in [23]. Briefly, a linear model was fitted to cow’s (N = 20,623 Holsteins) slopes for production trait (HTMYslope, HTFYslope, and HTPYslope that were pre-adjusted for the nongenetic effects described by [24]), for each autosomal SNP (~ 15 million SNPs). The model included a genomic relationship matrix (GRM) constructed based on the 50k SNP genotype data of the cows. The same model was used when performing GWAS for the multi-breed (Holstein and Jersey cows; N = 25,766) QTL discovery set except that an additional binary covariate was fitted to account for breed effect.

To increase the power of GWAS to identify pleiotropic variants for heat tolerance from the three slope traits, we combined the above single-trait GWAS results in a multi-trait meta-GWAS (following methods described in [30]), and described for the Holstein data set in [23].

Using either the single-trait or multi-trait GWAS results, we selected informative variants defined as ‘top SNPs’ for each slope trait as follows:

1. Moving along each chromosome in 100-kb sliding windows, we chose the single most significant SNP from within the 100-kb window and then moved 50 kb along the chromosome to the next 100-kb window. This was repeated starting from the proximal to the distal end of each chromosome, as in [11]. To be selected, the SNP had to pass a GWAS threshold of ${-\mathrm{log}}_{10}(\mathrm{p \,value})$ ≥ 3. In addition, we tested a more relaxed GWAS threshold of ${-\mathrm{log}}_{10}(\mathrm{p\, value})$ ≥ 2 to determine if it could help the capture of variants with much smaller effect sizes for heat tolerance in addition to those with large effects [23].
2. Among each set of selected ‘top SNPs’, we removed one SNP of any pair in strong linkage disequilibrium (LD) (r^2^ > 0.95) using the PLINK software [31], with the [−  indep-pairwise 50 5 0.95] option, where LD is calculated within 50-SNP sliding windows, each time sliding five SNPs along the chromosome.

### Genomic prediction using BayesR and BayesRC methods

We used BayesR [17, 32] to calculate genomic breeding values (GBV) for each cow in the validation set based on the standard-industry 50k SNP data. BayesR assumes one class of SNPs that are modelled as a mixture of four normal distributions corresponding to zero-, small-, medium- and large-sized effects [17, 32]. Currently, the Australian dairy industry uses the standard 50k SNP panel for routine genomic evaluations; thus, it served as the benchmark to test the added value of selected sequence variants (i.e., ‘top SNPs’). Furthermore, the standard-industry 50k SNP panel includes a set of variants that were not selected intentionally for heat tolerance, which was ideal for our study.

The BayesR model fitted 42,572 variants (SNPs with a MAF > 0.005) from the 50k SNP panel using bulls (N = 3323) as reference set:

1 $$\mathbf{y}=\mathbf{X}{\varvec{\upbeta}}+\mathbf{W}\mathbf{v}+\mathbf{e},$$

where $\mathbf{y}$ is a vector of heat tolerance slope phenotypes (HTMYslope, HTFYslope, and HTPYslope) or intercept (i.e., mean yield) traits (MYint, FYint, and PYint); $\mathbf{X}$ is a design matrix; ${\varvec{\upbeta}}$ is a vector of fixed effect solutions; $\mathbf{W}$ is a centred design matrix of SNP genotypes; $\mathbf{v}$ is a vector of SNP effects, modelled to have four possible normal distributions: $\mathbf{v} \sim N({\bf{0}}, {\mathbf{I}}{\sigma_i^2})$, where ${\sigma }_{i}^{2}=\left\{0.0, 0.0001*{\sigma }_{v}^{2}, {0.001*\sigma }_{v}^{2}, 0.01* {\sigma }_{v}^{2} \right\},$ corresponding to zero-, small-, medium- and large-sized effects, respectively with ${\sigma }_{v}^{2}$ the additive genetic variance; $\mathbf{e}$ is a vector of residual errors $N({\bf{0}},{\mathbf{E}}{\sigma }_{e}^{2})$, with $\mathbf{E}$ a diagonal matrix calculated as *diag*$(1/{w}_{i}$), with ${w}_{i}$ being a weighting factor for bull $i$ calculated based on the available number records following [33]:

2 $${w}_{{bull}_{i}}=\frac{1- {h}^{2}}{c{h}^{2}+ \frac{(4- {h}^{2})}{p}},$$

where ${h}^{2}$ is the heritability; $c$ is the proportion of the genetic variance that is not accounted by the SNPs ($c$ = 0.2); and $p$ is the number of daughters for each bull.

The same model (Eq. 1) was used when analysing the multi-breed reference population (Holstein and Jersey; N = 4175), except that a binary covariate was fitted to account for the breed effect. To account for polygenic effects, we tested models with or without pedigree relationships, which yielded correlation estimates of SNP effects close to 1.0. Therefore, based on these preliminary analyses, we decided not to include pedigree data in the subsequent models.

To calculate GBV using a combined set of 50k SNPs and the pre-selected SNPs from GWAS (i.e., 50k + ‘top SNPs’) for the validation cows, we used the BayesRC method [34]. BayesRC is an extension of BayesR in which two or more classes of SNP effects are modelled: the SNPs within each class are fitted as a mixture of four normal distributions as in BayesR so that the mixture distribution can differ for each SNP class. In our study, the SNPs from the standard 50k array (42,572 SNPs) were allocated to class I and the pre-selected ‘top SNPs’ from GWAS to a separate class II. Class I variants are considered as a random set from the 50k array (as indicated earlier), while Class II variants (‘top SNPs’) may be enriched with causal and/or highly predictive mutations for heat tolerance.

For both BayesR and BayesRC models, we performed five Markov chain Monte Carlo (MCMC) replicate chains, each with 40,000 iterations, of which 20,000 were discarded as burn-in for all the traits. These iterations gave stable convergence across the five replicates. The results from these replicates were averaged to get the final estimate. To facilitate the calculation of standard errors, we randomly split the validation cows into two subsets of approximately equal size (see Additional file 1: Table S2) and then performed analyses (i.e., the BayesR and BayesRC) for each subset, separately.

For each analysis (described above), the accuracy of prediction was calculated as described in [11]: $Accuracy= \frac{{r}_{GBV,phen}}{\sqrt{{h}^{2}}}$, where ${r}_{GBV, phen}$ is the correlation of GBV with TD phenotypes (slope or intercept traits); $({h}^{2}$ is the genomic heritability of the trait computed from 50k SNP data based on 29,107 Holstein cows). These heritability estimates used to calculate prediction accuracies are in Additional file 1: Tables S2 and S3. The corresponding standard errors of the accuracies were estimated as: $SE= SD/\sqrt{N}$, where $N$ is the number of random validation subsets ($N$ = 2); $SD$ is the standard deviation of the accuracies of prediction calculated from the two validation sets per breed (i.e., Holstein, Jersey, and crossbred cows). The dispersion bias of the accuracy of prediction for different traits was assessed as the regression coefficient of the TD phenotypes on the GBV in the validation set and their corresponding standard errors calculated as described for the $SE$ of the accuracies of prediction above. The regression coefficient = 1.0 indicates no dispersion bias, whereas values > 1.0 or < 1.0 indicate that the GBV are subject to deflation or inflation, respectively.

## Results

### Genomic heritability

Genomic heritability estimates based on 29,107 Holstein cows using the 50k SNP array were similar for all the slope (heat tolerance) traits (see Additional file 1: Table S2). The genomic heritability estimates based on Jersey cows (N = 6338) were comparable to those based on Holstein cows with values of 0.26 ± 0.02, 0.23 ± 0.02, and 0.25 ± 0.02 for the HTMYslope, HTFYslope and HTPYslope traits, respectively (see Additional file 1: Table S2). However, the values for crossbred cows (N = 790) were estimated with large standard errors [0.58 ± 0.10 (HTMYslope); 0.34 ± 0.11 (HTFYslope); 0.51 ± 0.10 (HTPYslope)], which is most likely due to the small sample size used. In contrast, the genomic heritability estimates for intercept traits were relatively larger than those for heat tolerance traits (see Additional file 1: Tables S2 and S3). In this study, we computed the accuracy of genomic predictions across all validation sets using the heritability estimates from Holstein cows (N = 29,107) that were estimated with the smallest standard errors.

### Pre-selection of heat tolerance SNPs (i.e., top SNPs)

#### Single-breed (Holstein cows) QTL discovery set

Table 1 includes the number of selected informative sequence variants for heat tolerance defined as ‘top SNPs’ from single-trait GWAS and multi-trait meta-analyses of the Holstein cow discovery set (i.e., the single-breed discovery set; see “Methods” section—“Scenario 1”). Using a more stringent GWAS cut-off threshold of − log10(p-value) ≥ 3 resulted in about a fivefold smaller number of selected ‘top SNPs’ than a comparatively relaxed GWAS cut-off of − log10(p-value) ≥ 2. The numbers of selected ‘top SNPs’ at a − log10(p-value) ≥ 2 from single-trait GWAS (after pruning pairs of markers in strong LD, r^2^ > 0.95) were equal to 9207 (HTMYslope), 9352 (HTFYslope), and 9633 (HTPYslope), and the numbers of those selected at a − log10(p-value) ≥ 3 were equal to 1654 (HTMYslope), 1708 (HTFYslope) and 1624 (HTPYslope) (Table 1). The largest number of ‘top SNPs’ was obtained for HTPYslope, followed by HTFYslope and HTMYslope (Table 1). Although the number of variants that passed the GWAS cut-off was largest for HTPYslope, the strength of the GWAS signal (peak) across the genome (see Additional file 2: Figs. S1 and S2) was relatively weak for this trait compared to the other traits (i.e., HTMYslope and HTFYslope).

**Table 1:** Number of informative markers for heat tolerance defined as ‘top SNPs’ selected from single-trait GWAS and multi-trait meta-analyses of heat tolerance slope traits of Holstein discovery cow set (N = 20,623)

Trait	‘Top SNPs’ (− log10(p-value) ≥ 2)	‘Top SNPs’ (− log10(p-value) ≥ 3)
HTMYslope	9207 (51,750)	1654 (44,219)
HTFYslope	9352 (51,894)	1708 (44,277)
HTPYslope	9633 (52,168)	1624 (44,190)
Meta-GWAS	9090 (51,636)	2365 (44,929)

Markers were selected based on the GWAS cut-off thresholds of − log10(p-value) ≥ 2 and − log10(p-value) ≥ 3. The values in brackets are the final number of SNPs after adding selected ‘top SNPs’ to the 50k SNP data used in the BayesRC analyses (i.e., 42,572 SNPs + top SNPs). Traits are defined as heat tolerance milk (HTMYslope), fat (HTFYslope) and protein (HTPYslope) yield slope traits

A large proportion (> 50%) of the selected ‘top SNPs’ had a lower MAF compared to the SNPs in the 50k panel (see Additional file 2: Fig. S3). Compared to single-trait GWAS, and as expected, fewer ‘top SNPs’ were selected from the multi-trait meta-analyses of slope traits at a more stringent [− log10(p-value) ≥ 3; N = 2365 SNPs] than at a relaxed GWAS cut-off [− log10(p-value) ≥ 2; N = 9090 SNPs] (Table 1). Comparatively, a slightly larger number of ‘top SNPs’ was selected across intercept traits than across heat tolerance traits (see Additional file 1: Table S4).

The proportion of phenotypic variance accounted for by the ‘top SNPs’ at a GWAS p-value cut-off of (− log10(p-value) ≥ 2; Table 1) varied across traits and populations. In general, the ‘top SNPs’ for HTMYslope explained a relatively larger variance compared to the ‘top SNPs’ for HTFYslope and HTPYslope across the studied scenarios. In the Holstein validation set, variance estimates for HTMYslope, HTFYslope and HTPYslope were 0.24 ± 0.05, 0.22 ± 0.05, and 0.21 ± 0.05, respectively. In the Jersey validation set, variance estimates explained by the ‘top SNPs’ for HTMYslope, HTFYslope and HTPYslope were 0.23 ± 0.02, 0.18 ± 0.02 and 0.22 ± 0.02, respectively. The variance estimates in the crossbred validation set were 0.55 ± 0.10, 0.24 ± 0.10, 0.37 ± 0.10, for HTMYslope, HTFYslope and HTPYslope, respectively. The large standard error for the variance estimates in crossbreds is likely due to their small sample size (N = 790).

#### Multi-breed (Holstein + Jersey cows) QTL discovery set

When Holstein cows (N = 20,623) were combined with Jersey cows (N = 5143) in the QTL discovery set (i.e., the multi-breed QTL discovery set, see “Methods” section—“Scenario 2”), we found a smaller number of selected ‘top SNPs’ (after pruning pairs of markers in strong LD, r^2^ > 0.95) from the single-trait GWAS at − log10(p-value) ≥ 2 [HTMYslope = 6132; HTFYslope = 6286; HTPYslope = 6422] compared to those from the single-breed QTL discovery set at the same significance cut-off (described above). However, when compared to the single-breed GWAS (only Holstein cows), using a multi-breed QTL discovery set (Holsteins + Jersey cows) increased the strength of the GWAS signals in some genomic regions (e.g., on *Bos taurus* chromosome (BTA) 14 near the *DGAT1* gene) (see Additional file 2: Figs. S4 and S5).

### Genomic prediction using selected SNPs from the single-breed discovery set (‘Scenario 1’)

**Fig. 2:**
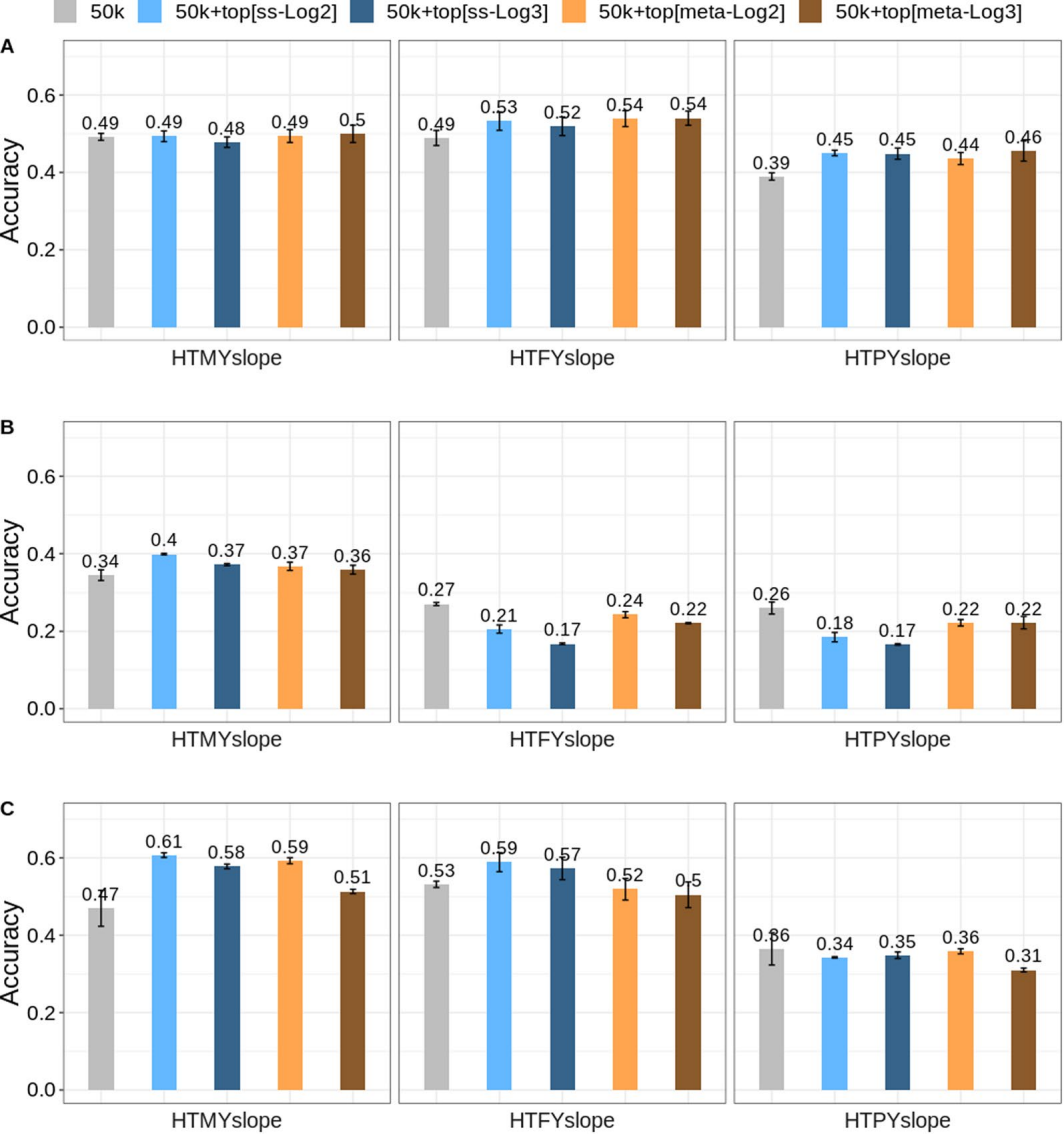
Accuracy of genomic predictions (Holstein only reference) using either 50k SNP data (colored grey) or 50k + a range of ‘top SNPs’ sets (selected from the Holstein QTL discovery set). The ‘top SNPs’ were selected from single-trait GWAS (colored blue) and multi-trait meta-analysis (colored orange) at a less stringent cut-off threshold of − log10(p-value) ≥ 2 [~ 9000 SNPs] and at a more stringent p-value of − log10(p-value) ≥ 3 [~ 2000 SNPs]. Accuracy of predictions are provided for three cow validation sets: **a** Holsteins, N = 1223), **b** Jersey, N = 6338), and **c** Holstein–Jersey crossbreds, N = 790). The traits analysed are heat tolerance milk (HTMYslope), fat (HTFYslope), and protein (HTPYslope) yield slopes. The genomic predictions were generated using either BayesR (50k SNP set) or BayesRC (50k + top SNPs). Vertical lines represent the standard errors calculated from two random validation subsets

Figure 2 shows the accuracy of predictions when the selected ‘top SNPs’ from a single-breed (Holstein cows; N = 20,623) QTL discovery set were added to the standard 50k SNP array and analysed using the BayesRC model. For this comparison, the reference set included only Holstein bulls (N = 3323) and the validation set included Holstein (N = 1223), Jersey (N = 6338) and crossbred (N = 790) cows. The gain in accuracy for the different traits and models varied across the three validation sets. The increase in the accuracy of prediction was generally consistent for HTMYslope across most of the different scenarios (50k + ‘top SNPs’) tested, but not for HTFYslope and HTPYslope, particularly in the Jersey validation set. In general, the increase in accuracy of prediction ranged from 0.001 to 0.09, with the largest increase (0.09) observed for HTMYslope in the crossbred validation set. In the Holstein validation set, the accuracy of prediction across all scenarios hardly changed for HTMYslope ranging from − 0.01 to 0.008 units, whereas the changes for HTFYslope and HTPYslope ranged from 0.03 to 0.05 and from 0.04 to 0.06, respectively (Fig. 2). For the intercept traits in the Holstein validation set, fitting 50k + ‘top SNPs’ (in the BayesRC model) generally increased the accuracy of prediction compared to BayesR (using only 50k SNPs) by up to 0.04, 0.03 and 0.05 for FYint, PYint and MYint, respectively (see Additional file 2: Fig. S6).

For the crossbred validation set, the change in the accuracy of prediction from BayesRC (50k + ‘top SNPs’) over BayesR (fitting only 50k SNPs) ranged from − 0.004 to 0.09, from − 0.06 to 0.02, and from − 0.04 to 0.009 for HTMYslope, HTFYslope, and HTPYslope, respectively (Fig. 2). Similarly, compared to BayesR (using only 50k SNPs), the accuracy of prediction for the intercept traits in crossbreds hardly changed across most prediction scenarios when fitting 50k + ‘top SNPs’ (BayesRC), with changes ranging from − 0.01 to 0.01, from − 0.02 to 0.02, and from − 0.04 to − 0.02 for MYint, FYint, and PYint, respectively (see Additional file 2: Fig. S6).

In the Jersey validation set (using 50k + ‘top SNPs’ in the BayesRC), we observed that the accuracy for HTMYslope increased compared to that of BayesR (using only 50k SNPs) across all prediction scenarios, with changes ranging from 0.01 to 0.05 units. However, the accuracy of prediction decreased considerably for HTFYslope (− 0.10) and HTPYslope (− 0.09) when the ‘top SNPs’ from Holstein cows were used in Jerseys with a slightly larger decrease in accuracy of prediction when using ‘top SNPs’ from the single-trait GWAS than those from the multi-trait meta-analysis (Fig. 2). Similarly, compared to BayesR using only 50k SNPs, the accuracy of prediction dropped for intercept traits when fitting the ‘top SNPs’ from the Holstein cow discovery set in Jerseys with changes ranging from − 0.10 to − 0.03 (FYint) and from − 0.09 to − 0.04; (PYint), while the accuracy of prediction for MYint from BayesRC increased from 0.02 to 0.06 compared to BayesR (see Additional file 2: Fig. S6).

Across all prediction scenarios (Fig. 2), using ‘top SNPs’ from the relaxed GWAS cut-off value of [− log10(p-value) ≥ 2 (~ 9000 SNPs) in the BayesRC model did not yield a substantial difference in accuracy of prediction compared to those based on the ‘top SNPs’ from a more stringent GWAS threshold [− log10(p-value) ≥ 3 (~ 2000 SNPs). The change in accuracy of prediction across all validation sets and traits ranged from − 0.07 to 0.09 units and from − 0.10 to 0.06 units when the ‘top SNPs’ from relaxed and more stringent GWAS cut-off p-values were added to the 50k SNP panel (BayesRC) compared to the results from BayesR (using only 50k) (Fig. 2). In general, using ‘top SNPs’ from the more stringent GWAS cut-off in the BayesRC model yielded a larger dispersion bias than the ‘top SNPs’ from the relaxed GWAS cut-off threshold for heat tolerance slope traits (see Additional file 2: Fig. S7). However, for intercept traits, the BayesRC model using 50k + top SNPs showed little or no increase in the dispersion bias (see Additional file 2: Fig. S8).

Moreover, there was no substantial difference in accuracy from BayesRC when using the ‘top SNPs’ from single-trait GWAS versus the ‘top SNPs’ from multi-trait meta-GWAS of slope traits across different prediction scenarios (Fig. 2). The change in accuracy based on the selected ‘top SNPs’ from the single-trait GWAS ranged from 0.002 (HTMYslope) to 0.06 (HTPYslope), from − 0.05 (HTFYslope) to 0.02 (HTMYslope), and from − 0.06 (HTFYslope) to 0.07 (HTMYslope) in Holsteins, Jerseys, and crossbred validation sets, respectively. These changes are comparable to those obtained using ‘top SNPs’ from the meta-analysis of slope traits with changes ranging from − 0.01 (HTMYslope) to 0.06 (HTPYslope), from − 0.10 (HTFYslope) to 0.05 (HTMYslope), and from − 0.006 (HTPYslope) to 0.09 (HTMYslope) in Holsteins, Jersey, and crossbred validation sets, respectively. Since the results were comparable when using ‘top SNPs’ from either relaxed or stringent GWAS cut-off values, we hereafter, only report the results based on the ‘top SNPs’ from the single-trait GWAS at the relaxed cut-off threshold (i.e., − log10(p-value) ≥ 2).

The dispersion bias across heat tolerance traits in the Holstein validation set ('Scenario 1') showed that the GBV were deflated (see Additional file 2: Fig. S7). In contrast, the predictions were less biased (i.e., regression coefficient values closer to 1.0) for the intercept traits, particularly for MYint and PYint in the Holstein validation set (see Additional file 2: Fig. S8). In the Jersey validation set, the dispersion bias for HTFYslope showed that the GBV were inflated (see Additional file 2: Fig. S7). Also, the GBV were inflated in the Jersey validation set for HTPYslope when the ‘top SNPs’ were added to the 50k SNP array and analysed using BayesRC [i.e., 1.11 in the BayesR model versus 0.79 in the BayesRC model; (see Additional file 2: Fig. S7)]. The dispersion bias for the Jersey validation set was inflated across intercept traits (see Additional file 2: Fig. S8). The predictions were extremely deflated in the crossbreds, particularly for HTMYslope (bias > 1.7), which is likely due to the small sample size and population used. The dispersion bias for heat tolerance traits was even more pronounced when the selected ‘top SNPs’ were added to the 50k SNP data using the BayesRC model compared to the estimates using the BayesR model and only the 50k SNP data (see Additional file 2: Fig. S7).

### Genomic prediction using selected SNPs from multi-breed discovery set (‘Scenario 2’)

**Fig. 3:**
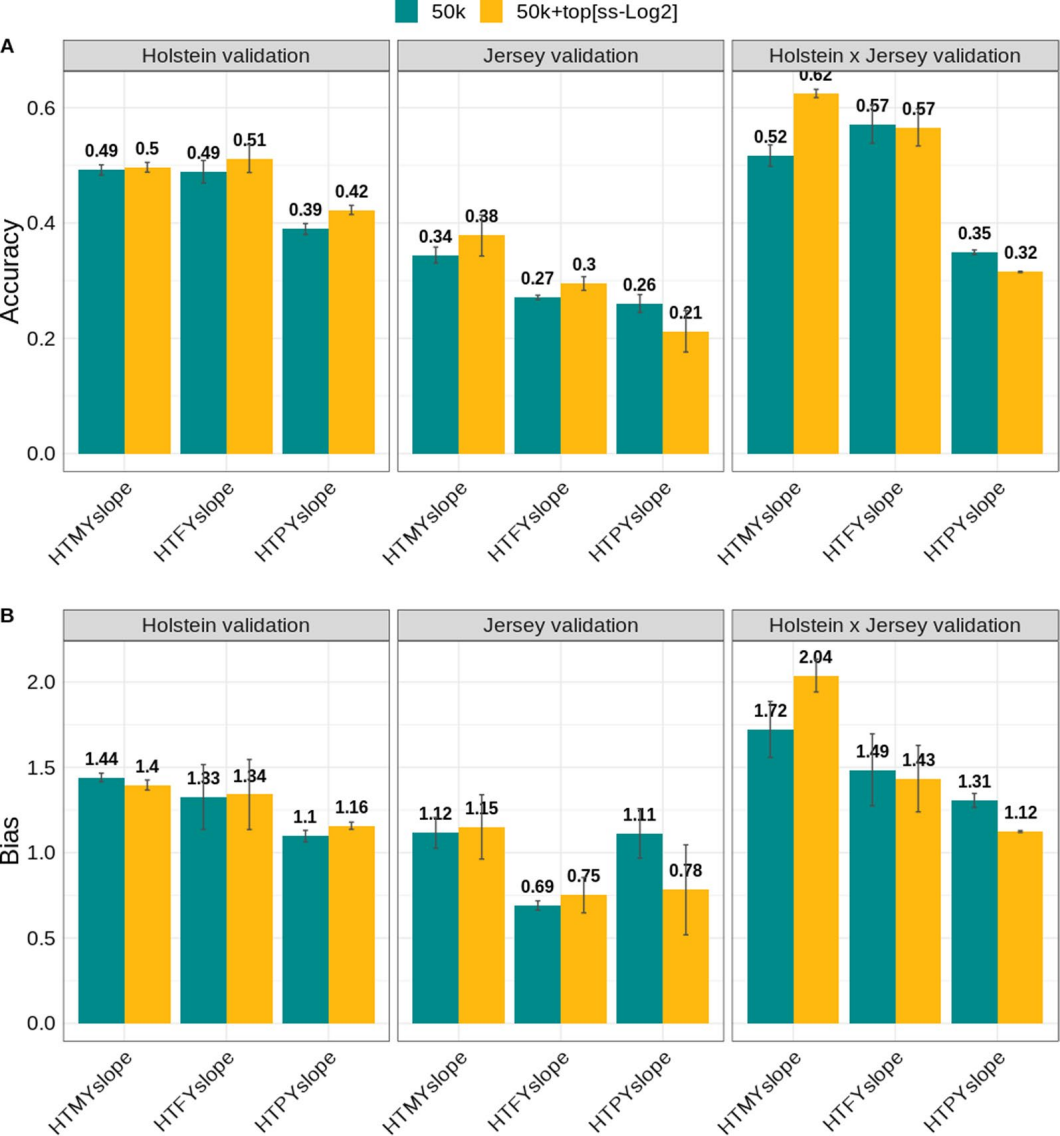
Accuracy and dispersion bias in Holstein (N = 1223), Jersey (N = 1195) and crossbred (N = 790) cows when using 50k + ‘top SNPs’ selected from the multi-breed (Holstein + Jersey) QTL discovery set. Holstein bulls (N = 3323) were used as the reference set for genomic predictions. The ‘top SNPs’ were selected based on a single-trait GWAS cut-off of [− log10(p-value) ≥ 2]. The traits analysed are heat tolerance milk (HTMYslope), fat (HTFYslope), and protein (HTPYslope) yield. Vertical lines represent the standard errors calculated from two random validation subsets

Figure 3 shows the change in accuracy of prediction (based on the BayesRC) when the selected ‘top SNPs’ (GWAS cut-off of − log10(p-value) ≥ 2) from the multi-breed (Holstein + Jersey cows) QTL discovery set were added to the 50k SNP array for which the reference set consisted only of Holstein bulls. In general, the change in accuracy of prediction across all traits and validation sets ranged from − 0.05 (HTPYslope) in Jersey to 0.11 (HTMYslope) in crossbred cows. In the Holstein validation set (N = 1223), the accuracy of prediction increased across all traits with the greatest increase for HTPYslope (0.03) followed by HTFYslope (0.02) and HTMYslope (0.005), respectively. In this validation set, the dispersion bias was higher than 1.0 across all traits, indicating deflated GBV. The bias decreased slightly for HTMYslope but increased for HTPYslope and HTFYslope when the ‘top SNPs’ were fitted in the BayesRC model (Fig. 3).

In the Jersey validation set (N = 1195), the change in accuracy of prediction (based on the BayesRC model) was not consistent across traits (Fig. 3). When using the selected ‘top SNPs’ from the multi-breed QTL discovery set, the accuracy of prediction increased for HTMYslope (0.03) and HTFYslope (0.02) but decreased for HTPYslope (− 0.05). These values contrast with those obtained using selected ‘top SNPs’ from the single-breed QTL discovery set (only Holsteins; see “Methods”, “Scenario 1”), where we found a change in accuracy of 0.09, 0.04, and 0.01 for HTMYslope, HTFYslope, and HTPYslope, respectively, when using a smaller subset of Jersey cows (i.e., N = 1195) instead of 6338 cows (as in “Scenario 1”). Unlike in “Scenario 1” where we found that GBV were inflated across heat tolerance traits (see Additional file 2: Figure S7) in Jerseys, the predictions were generally close to 1.0 in “Scenario 2”, particularly for HTMYslope (Fig. 3).

In the crossbreds (N = 790), using ‘top SNPs’ discovered in the multi-breed (Holsteins + Jersey cows) set (based on the BayesRC models) yielded a larger (0.11 units) change in accuracy of prediction than with BayesR (using only 50k SNPs) for HTMYslope compared to a drop in accuracy from BayesRC over BayesR of − 0.005, and − 0.03 units for HTFYslope and HTPYslope, respectively (Fig. 3). Comparatively, using the ‘top SNPs’ from the single-breed (only Holsteins) QTL discovery set in crossbreds (‘Scenario 1’) yielded a change in accuracy from BayesRC over BayesR of 0.09, 0.02, and − 0.006 for HTMYslope, HTFYslope, and HTPYslope, respectively. As in “Scenario 1”, the dispersion bias in crossbreds for HTMYslope was extreme (> 1.7) compared to the other traits. In this crossbred validation set (‘Scenario 2’), the bias increased more for HTMYslope but decreased for HTFYslope and HTPYslope when fitting the selected ‘top SNPs’ in BayesRC (Fig. 3).

### Genomic prediction using multi-breed reference set (‘Scenario 3’)

**Fig. 4:**
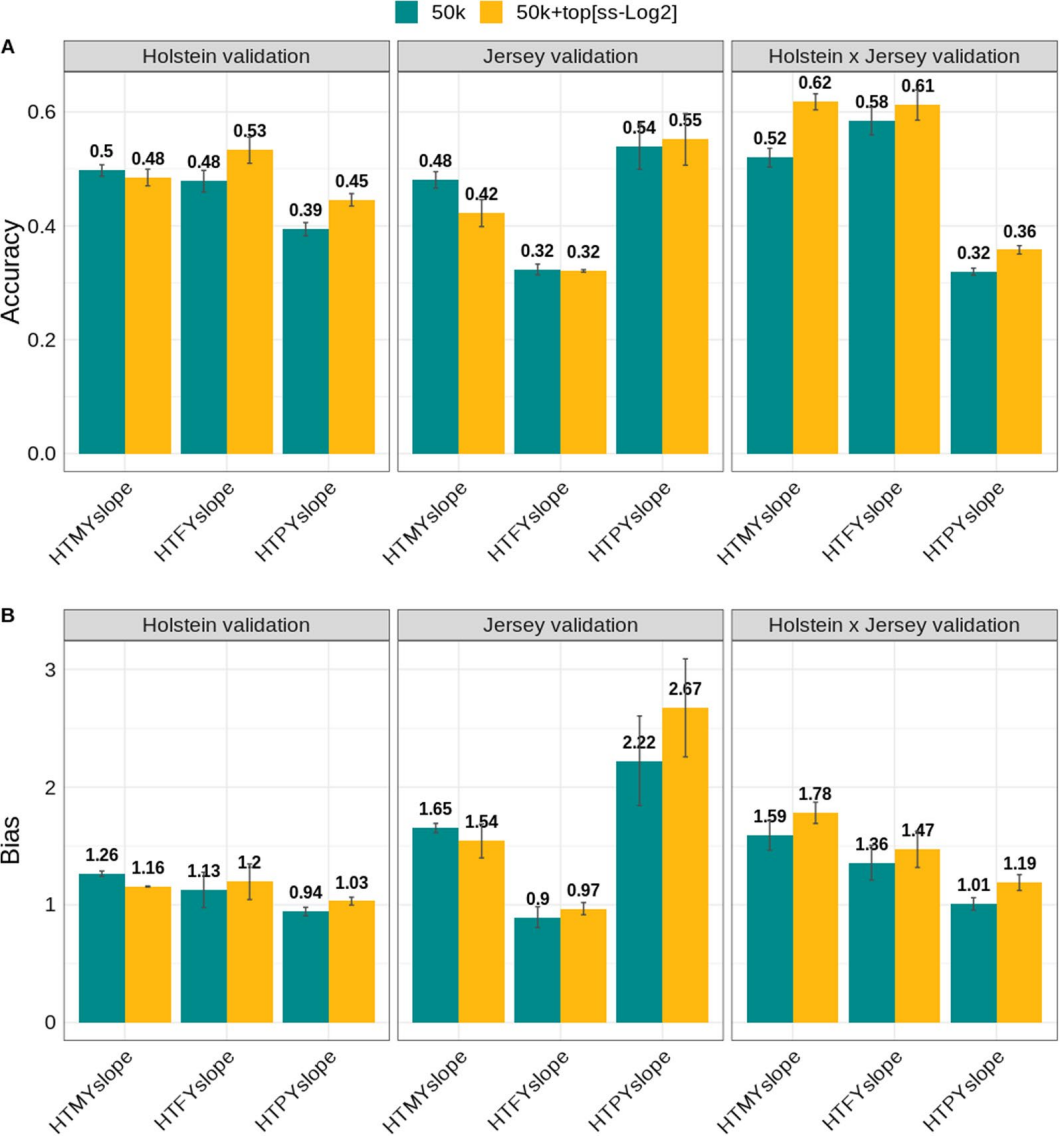
Accuracy and bias of genomic predictions in Holstein (N = 1223), Jersey (N = 431) and crossbred (N = 790) cows when using the multi-breed reference set (Holstein and Jersey bulls; N = 4175). The selected ‘top SNPs’ used in the BayesRC were from the Holstein cow discovery set (N = 20,623) based on the single-trait GWAS cut-off of [− log10(p-value) ≥ 2]. The traits analysed are heat tolerance milk (HTMYslope), fat (HTFYslope), and protein (HTPYslope) yield slopes. Vertical lines represent the standard errors calculated from two random validation subsets

When we used a multi-breed (Holstein + Jersey bulls) reference set in which the ‘top SNPs’ were only from the Holstein cow QTL discovery set (see “Methods” section, “Scenario 3”), we found a consistent increase in the accuracy of prediction in most cases (Fig. 4). The accuracy of prediction decreased only for HTMYslope (− 0.06) and HTPYslope (− 0.002) in the Jersey validation set for this scenario. The change in accuracy of prediction from BayesRC over BayesR for HTMYslope, HTFYslope, and HTPYslope were: [− 0.01, 0.05, and 0.05], [− 0.06, − 0.002, and 0.01], and [0.10, 0.03, and 0.04] in the Holstein (N = 1223), Jersey (N = 431) and crossbred (N = 790) cow validation sets, respectively (Fig. 4). These changes in accuracy of prediction are slightly larger compared to those found when using a single-breed reference set (Fig. 2; “Scenario 1”) from BayesRC over BayesR, with values for HTMYslope, HTFYslope and HTPYslope of [0.001, 0.04, and 0.06], [0.05, − 0.06 and − 0.07], [0.09, 0.02, and − 0.006] in the Holstein (N = 1223), Jersey (N = 6338), and crossbred (N = 790) validation sets, respectively. To be more comparable, when considering only a subset of Jersey cows (N = 431) in the validation set where the reference set consisted of a single breed (only Holstein bulls; “Scenario 1’), we found a change in accuracy from BayesRC over BayesR of − 0.02, 0.03, and − 0.06 for HTMYslope, HTFYslope, and HTPYslope, respectively. Compared to estimates from the “Scenario 1” and “Scenario 2” analyses above, we observed the smallest bias (i.e., values around 1.0) when using the multi-breed reference set in the Holstein validation set. However, in the Jersey validation set, we found extreme bias (> 2.0) for HTPYslope, whereas the bias was smaller for HTFYslope. In the crossbreds, the bias was large for HTMYslope (> 1.5) and HTFYslope (> 1.3), whereas we observed a small bias (values closer to 1.0) for the HTPYslope trait.

### BayesR versus BayesRC methods

To test whether allocating selected informative markers to a separate SNP class (see “Methods”) in BayesRC can show added benefit in our study, we combined 50k + ‘top SNPs’ from a single-breed (Holsteins) QTL discovery set and re-calculated GBV using BayesR where all SNPs were allocated to a single class. The total number of 50k + ‘top SNPs’ used in BayesR and BayesRC was 51,750, 51,894, and 52,168, for HTMYslope, HTFYslope, and HTPYslope traits, respectively (Table 1). The accuracy of prediction (± SE) was slightly higher for two of the three traits from BayesRC [0.49 ± 0.01 (HTMYslope); 0.53 ± 0.02 (HTFYslope); 0.45 ± 0.007 (HTPYslope)] compared to BayesR [0.51 ± 0.01 (HTMYslope); 0.51 ± 0.02 (HTFYslope); 0.44 ± 0.01 (HTPYslope)]. These results suggest that allocating SNPs to different classes in BayesRC, yields marginal benefit in the prediction of heat tolerance traits over BayesR. Moreover, there was little difference in the regression coefficient of predictions (± SE) between BayesRC [1.42 ± 0.002 (HTMYslope); 1.31 ± 0.08 (HTFYslope); 1.21 ± 0.02 (HTPYslope)] and BayesR [1.33 ± 0.005 (HTMYslope); 1.32 ± 0.17 (HTFYslope); 1.21 ± 0.02 (HTPYslope)].

## Discussion

In this paper, we present a genomic prediction analysis of heat tolerance traits using a large sample size of over 40,000 cattle, comprising Holstein, Jersey, and crossbred individuals. The primary objective was to investigate if selected sequence variants from a GWAS in Holstein cattle benefits genomic prediction of heat tolerance phenotypes in the same breed (i.e., within-breed prediction). The hypothesis is that the selected variants are linked to causal mutations that underpin the genetic basis of heat tolerance, and thus could enable more accurate and sustained genomic selection for this trait. In addition, we also tested the value of pre-selected variants from Holstein cattle for the genomic prediction of breeds with numerically smaller sample sizes, such as Jersey and crossbreds. Furthermore, we investigated the benefits of using informative markers from a multi-breed (Holstein + Jersey cows) QTL discovery set for genomic prediction of heat tolerance. Overall, our results show that we can increase the accuracy of prediction of heat tolerance by up to 0.10 unit in some scenarios when pre-selected sequence variants are added to the standard-industry 50k SNP panel. However, the change in the accuracy of prediction when using pre-selected sequence variants in BayesRC (i.e., 50k + top SNPs) varied considerably across traits and prediction scenarios.

We used the BayesR and BayesRC methods to test different prediction scenarios. For BayesR, when only 50k SNP data were used, we found a high accuracy of prediction in Holsteins and crossbreds compared to Jerseys. We expected a lower accuracy in Jerseys because we used Holstein bulls as a reference set for genomic predictions (see “Methods”, “Scenario 1”). These breeds are genetically divergent and may differ regarding the linkage disequilibrium of variants with causal mutations, they may not share all the same causal variants, or some variant effects may differ between these breeds [35]. As such, when we combined Holstein and Jersey bulls in the reference set (multi-breed reference set; see “Methods”, “Scenario 3”) and performed analysis using BayesR (without pre-selected ‘top SNPs’), we found a substantial improvement in the accuracy of prediction across all traits for Jerseys which is consistent with the multi-breed genomic predictions reported in previous studies e.g., [17, 35].

For the BayesRC model, where 50k + selected ‘top SNPs’ were fitted in the analysis, we found a consistent increase in the accuracy of prediction across traits when using the ‘top SNPs’ that were selected from the Holstein discovery set for the prediction of the Holstein validation cows (i.e., within-breed QTL discovery and validation set; see “Methods”, “Scenario 1”). Similarly, the use of ‘top SNPs’ from the Holstein discovery set in crossbred cattle based on BayesRC performed reasonably well, as expected since our crossbred cows share a similar genetic background with Holsteins (i.e., there were mostly F_1_ and backcrosses to Holstein). The gain in accuracy of prediction for Holsteins and crossbreds likely benefited, in part, from a powerful GWAS QTL discovery set (we used a sample size of 20,623 Holstein cows, each having around 15 million imputed sequence variants) and the methodology used for genomic prediction. To date, comparable GWAS have used a sample size of at most 5000 individuals e.g., [5] to search for variants linked to heat tolerance in dairy cattle. We expect an even greater increase in accuracy of prediction in the future with larger sample sizes for GWAS to increase the power of QTL discovery.

However, the genomic predictions in Jerseys performed rather poorly, particularly for HTFYslope and HTPYslope, with accuracies decreasing when the selected ‘top SNPs’ from the Holstein discovery set were added to the 50k SNP set and used in BayesRC. Given that Holstein and Jersey are genetically divergent breeds, using informative QTL from Holstein in a Jersey validation may have introduced noise in the genomic predictions since the common QTL may not be tracked across these breeds. Also, the drop in accuracy could be due to the non-additive genetic effects (i.e., dominance and epistasis) between Holstein and Jersey. Simulation studies e.g., [36] found that the additive genetic correlations between divergent populations can drop to values as low as 0.45 if reasonably large epistatic interactions exist among loci, which can impact genomic predictions across populations.

However, it is rather unclear why the accuracy of prediction increased for HTMYslope in Jerseys but not for HTFYslope and HTPYslope when using selected ‘top SNPs’ from Holsteins. One reason could be due to a difference in genetic architecture of these traits. One way to explain this result is to examine the direction of effect for the SNPs between populations. For example, by imposing the GWAS cut-off p-value of 0.001 in both Holstein bulls and Jersey cows, we found that 72% (N = 774) and 71% (N = 524) of the effects for the significant SNPs for HTMYslope and HTFYslope, respectively, were in the same direction. Comparatively, we found a larger proportion of significant SNPs (GWAS p-value < 0.001) having the same direction of effects for HTMYslope (85%; N = 420) and HTFYslope (95%; N = 1240) between Holstein bulls versus Holstein cows (i.e., within-breed comparison) (see Additional file 1: Table S5). Besides the direction of effects for the SNPs between populations, a smaller number of ‘top SNPs’ for HTMYslope was discovered from the GWAS in Holstein cattle at the relaxed cut off (p < 0.01) (Table 1) compared to HTPYslope and HTFYslope, suggesting that HTMYslope is controlled by relatively few QTLs with large effects compared to the other traits. This is supported by the strength [based on the magnitude of − log10(p-value)] and the number of significant GWAS signals across the genome based on the Manhattan plot (see Additional file 2: Figure S1). For HTMYslope, we observed four strong peaks on four chromosomes (i.e., BTA5, 6, 14, and 20). This contrasts with the HTFYslope trait for which we observed multiple clear GWAS signals across the genome (see Additional file 2: Figure S1). Moreover, these results are consistent with the evidence that the ‘top SNPs’ for the HTMYslope trait explained a relatively larger proportion of phenotypic variance compared to the ‘top SNPs’ for other traits across prediction scenarios.

By comparing the GWAS in Holsteins (N = 20,623) and Jersey (N = 6338) cows, we found the greatest overlap of top significant SNPs (i.e., top SNPs that were at least within 1-Mb regions in both breeds) for HTMYslope mapping to the genomic regions showing strong signals on BTA5, 14, 20, and 25. This overlap explains, in part, the greater consistency of the increase in accuracy of prediction for HTMYslope than for HTFYslope and HTPYslope. In this context, our findings are in line with those of [32], who reported that only a fraction of the QTL for milk yield segregate across Holstein and Jersey cattle. Overall, these results suggest that breed × SNP interactions exist, meaning that the informative markers obtained from Holstein are of little or no value for the prediction in Jersey. These findings have implications in the genomic prediction of complex traits such as heat tolerance since it is not unusual for one country to incorporate genetic variants discovered in an independent study from another country in their genomic evaluations, e.g., a meta-analysis of SNP effects from multiple countries using SNP-multiple across country evaluation (MACE) [37]. In addition, the results in this study seem to indicate that HTMYslope could be a more reliable indicator trait of heat tolerance and could be given greater weight in the selection index that incorporates heat tolerance, although further work is needed to confirm this. Currently, the Australian dairy industry gives more economic weight to HTPYslope (6.92) than to HTMYslope (− 0.10) or HTFYslope (1.79) in the calculation of heat tolerance genomic breeding values based on weights for milk production traits [38, 39].

Previous research studies in cattle e.g., [18, 19] have reported that the mapping of putative causal mutations is more precise when using multi-breed populations in GWAS and have proposed pathways that underpin heat tolerance [23]. In this study, we found some improvement in the predictions, especially in Jersey, when using ‘top SNPs’ from a discovery set of combined Holstein and Jersey cows (i.e., the multi-breed QTL discovery set). For example, the accuracy of prediction increased by 0.03 for HTFYslope when using ‘top SNPs’ selected from the multi-breed discovery set in Jersey compared to a drop of 0.06 when the ‘top SNPs’ from the Holstein QTL discovery set (single breed) was used in BayesRC (Fig. 3). In principle, combining divergent breeds in the QTL discovery set may help to break long-range LD, such that the selected ‘top SNPs’ are closer to the causal mutations [17] than when a single-breed QTL discovery set is used. For example, the top significant SNP on BTA14 mapped to the upstream region of the *SLC52A2* gene and in an intron of the *HSF1* gene when using the single-breed and multi-breed QTL discovery sets, respectively (see Additional file 2: Figure S9). The *HSF1* gene is associated with thermotolerance in dairy cattle [5, 6, 23]. The smaller number of ‘top SNPs’ detected in our study with the multi-breed than with the within-breed QTL discovery set is consistent with the work of [19] and is attributed, in part, to the causal variants not all segregating across the Holstein and Jersey breeds.

However, we could still see a decrease in accuracy of prediction (− 0.05) for HTPYslope when using the ‘top SNPs’ from a multi-breed discovery set in Jersey, although not as high as that (− 0.08 units) found when using the ‘top SNPs’ from the single-breed (Holsteins) discovery set. As discussed earlier, one reason for the observed poor prediction for these traits in Jerseys could be partly due to the breed × SNP interactions or non-additive epistatic interactions among loci across breeds. Notably, our multi-breed QTL discovery set was highly dominated by Holstein individuals which explains, in part, the limited gain in accuracy when the selected ‘top SNPs’ from the multi-breed discovery set were used in the Jerseys. Besides, we used Holstein bulls as a reference set in genomic predictions in Jerseys. Since these breeds are divergent, a better approach to improve predictions in Jerseys would have been to use ‘top SNPs’ from a multi-breed or within-breed (Jersey) QTL discovery set and a reference set of the same breed (Jersey) or multi-breed set. However, compared to Holstein, the smaller number of Jersey individuals in our study means that it was not possible to split the Jersey dataset to obtain independent subsets with sufficient power for use in the QTL discovery and reference set for genomic predictions. This implies that there may be more room for improvement in accuracy of prediction for Jerseys when more animals with phenotype and genotype data are available in the future.

We compared the added value of informative markers (i.e., ‘top SNPs’) from single-trait GWAS versus multi-trait meta-GWAS in the genomic predictions. The aim of the meta-analysis of slopes was to increase the power of GWAS and obtain a set of ‘top SNPs’ with putative pleiotropic effects for heat tolerance phenotypes. There is a recent trend towards developing custom SNP arrays that include variants with pleiotropic effects across multiple traits [40, 41]. In this study, we found a comparable increase in accuracy of prediction when we used ‘top SNPs’ from single-trait GWAS or from the meta-analysis, although the accuracy of prediction varied considerably across traits and validation sets used (Fig. 2). Our recent work [23] suggests that heat tolerance traits (milk, fat, and protein slopes) are regulated differently in heat-stressed dairy cows. As such, we think that the relatively lower accuracy realized from using selected ‘top SNPs’ from the meta-GWAS of slope traits in some scenarios (e.g., HTMYslope across the three validation sets; Fig. 2) could be due to the possible inclusion of non-causal ‘top SNPs’ in genomic prediction, which arose from combining SNP effects for different heat tolerance phenotypes. However, we observed a smaller drop in accuracy of prediction when using ‘top SNPs’ from the meta-GWAS compared to ‘top SNPs’ from the single-trait GWAS in Jerseys from the BayesRC over BayesR (Fig. 2).

In general, we demonstrated an increase in the accuracy of prediction of heat tolerance when informative sequence markers were added to the 50k SNP panel by up to 0.07, 0.05, and 0.10 units in Holstein, Jersey, and crossbred cows in some cases, respectively. Our findings are within the range of those reported for complex traits in cattle e.g., [42] and sheep e.g., [11, 13]. For example, Al Kalaldeh et al. [13] reported an increase in accuracy of prediction by 0.09 units for parasitic resistance in Australian sheep, while de Las Heras-Saldana et al. [42] found an increase of up to 0.06 units for carcass traits in cattle. These results indicate that informative markers can be prioritised, especially for the development of customized SNP arrays [41]. Adding informative variants for heat tolerance to the custom SNP panels as in [41] ensures that higher accuracies of prediction are achieved, which will help to drive genetic gain for this trait. Moreover, we expect that the genetic prediction of this trait would be sustained over generations when informative variants that are closer to the causal mutations are included in the custom SNP panels, as demonstrated by Khansefid et al. [43]. These authors found that using the custom XT_50k SNP panel, which contains prioritised sequence markers, gave a consistent and superior accuracy of prediction (compared to standard SNP panels) in crossbred cows (crossbreds represents “more distant relationships or many generations”). However, caution is needed when using pre-selected sequence variants from Holsteins in the prediction of Jerseys, considering that we found a decrease in accuracy, in most cases, when using the ‘top SNPs’ from the Holstein discovery set in Jerseys (Fig. 2). This agrees with the simulation work by [14] who reported that the decrease in accuracy of predictions across-breeds depended on the distance between causal mutations and the markers.

Some studies, e.g., [11, 13], using genomic best linear unbiased prediction (GBLUP), have reported increased accuracy of prediction when fitting pre-selected sequence variants from GWAS together with standard 50k SNPs compared to fitting only standard 50k SNPs, especially when modelling separate GRM for 50k SNPs and pre-selected SNPs. To compare our results (from the BayesR and BayesRC analyses), we used GBLUP on the Holstein validation set (as in “Scenario 1”; see “Methods”) to fit pre-selected heat tolerance SNPs (‘top SNPs’) either as one GRM (i.e., combined set of 50k + top SNPs) or separate GRM (i.e., 2 GRM) for 50k and ‘top SNPs’. Although the accuracy of prediction increased when fitting two GRM compared to fitting only one GRM in the GBLUP model, BayesR and BayesRC outperformed GBLUP for the prediction of HTMYslope and HTFYslope but not for that of HTPYslope (see Additional file 1: Table S6) and see Fig. 2. This is comparable to the work of [44] who reported better predictions for milk yield and fat yield traits from Bayesian models than GBLUP models in Danish cattle.

In this study, a sizable proportion of the selected ‘top SNPs’ for heat tolerance (slopes) overlapped with the selected ‘top SNPs’ for the intercept traits: 11% (HTMYslope), 17% (HTFYslope) and 21% (HTPYslope) (see Additional file 1: Table S7). Notably, when assuming QTL windows of 1 Mb, more than 90% of the selected ‘top SNPs’ for heat tolerance traits fell within the same windows with those for intercept traits, which is consistent with the high (− 0.80) phenotypic correlations between these traits. In our recent work [23], we demonstrated, through conditional GWAS analyses, that the top GWAS hits/signal for heat tolerance are also important for milk production traits (i.e., intercept). Therefore, a key question is whether using the selected sequence variants for heat tolerance in genomic evaluations can impact milk production. We investigated this assuming (1) selection is for milk production traits (or Australian Selection Index (ASI), i.e., traits are weighted according to the way Australian farmers are paid for milk, fat, and protein) and (2) selection is for the balanced performance index (BPI) which includes production and functional traits [39]. To see the impact of using pre-selected SNPs in genomic evaluations of Holsteins, the correlation between EBV for heat tolerance (estimated with only 50k SNPs or 50k + selected sequence variant from GWAS—‘top SNPs’) and ASI or BPI values were used. The correlation estimates (see Additional file 1: Table S8) suggest that adding the pre-selected SNPs for heat tolerance (including those that overlapped with intercept traits) to the standard-industry 50k array has little to no impact on the ASI and BPI. However, we observed a favourable correlation between heat tolerance and BPI when pre-selected ‘top SNPs’ from HTMYslope are added into the 50k array (i.e., 0.06 (50k) versus 0.10 (50k + meta-GWAS top SNPs; see Additional file 1: Table S8). These results are comparable to those of [7], who found that the current selection practices in Australia based on BPI will lead to a negligible decrease in heat tolerance (measured as the rate of decline in yield).

In addition, some reports e.g., [3, 4] have raised concerns that selection for heat tolerance may negatively impact the genetic progress for milk production due to a strong genetic correlation of about − 0.80 between these traits [23]. Notably, the effects of all the overlapping SNPs for HTMYslope (see Additional file 1: Table S7) were in the same direction with those for MYint, whereas the effects of the overlapping SNPs for HTFYslope and HTPYslope were in opposite directions with their corresponding intercept traits (i.e., FYint and PYint). However, the overlap of top SNPs for MYint and HTMYslope is only 11% (see Additional file 1: Table S7).

Most of our dispersion bias of prediction for heat tolerance traits from BayesR and BayesRC were deflated. However, we also observed inflated predictions, in some cases, especially in Jerseys. In all our Bayesian analyses, we used only bulls in the reference population and only cows in the validation of genomic predictions. As such, the smaller variance of bull phenotypes resulting from averaging daughter slope solutions (see “Methods”) explains, in part, the observed bias, especially in the Holstein cow validation set. To test this, we split Holstein cows into reference (older cows) and independent validation (young cows) sets. Consequently, we found that the GBV were inflated, which supports our hypothesis. Nevertheless, the magnitude of bias observed in this study may not be a big issue in the genomic evaluations of heat tolerance, where breeding values are calculated jointly based on bull and cow phenotypes using different weightings according to the amount of information [7, 38].

By comparing the Bayesian (BayesR and BayesRC) versus the GBLUP models (fitting either 1 or 2 GRM as described earlier), we found slightly less biased predictions from the former than the latter models (see Additional file 1: Table S6). This was expected since the Bayesian models simultaneously account for all the markers in the analysis and assume different distributions of SNP effects. However, recent studies in sheep [11] and cattle [43] have reported no difference in dispersion bias between the BayesR or emBayesR versus GBLUP models. We also assessed the dispersion bias of prediction for heat tolerance traits from the GBLUP and BayesR models using the linear regression (LR) method described by Legarra and Reverter [45]. We did this by first estimating SNP effects from: (1) the full (N = 3323 ♂) Holstein reference set, and (2) a randomly selected reduced (N = 1662 ♂; 50%) reference set. We found no dispersion bias when regressing the GBV in the Holstein validation cows (N = 1223) generated from the full reference bull set on the GBV from the reduced reference bull set (see Additional file 1: Table S9). This suggests that the SNP effects from these reference sets are robust in terms of genomic predictions.

The fact that the dispersion bias of prediction, in most cases, was more pronounced when the selected ‘top SNPs’ were added to the 50k SNP array and analysed with BayesRC is consistent with some previous studies [20, 21], which is likely due to a phenomenon called the “Beavis effect” [46] that originates from the overestimation of the effect size of the pre-selected variants. The lower bias found when fitting the selected ‘top SNPs’ from the stringent GWAS cut-off than from the relaxed GWAS cut-off does not agree with the results of Veerkamp et al. [21], who reported a larger bias when markers were strongly pre-selected. Here, we used the Bayesian approach (BayesRC), while Veerkamp et al. [21] applied GBLUP. In our study, fitting separate GRM for the 50k and the selected ‘top SNPs’ (i.e., two GRM) in the GBLUP models reduced the dispersion bias compared to fitting only one GRM for the 50k + top SNPs (see Additional file 1: Table S6).

In this study, we investigated the utility of pre-selected sequence variants in the genomic prediction of heat tolerance for milk production traits (milk, fat, and protein yield). It is also worthwhile to investigate the added value of prioritised sequence variants for heat tolerance on other traits that are affected by heat stress (e.g., fertility) because there are likely to be benefits from achieving higher systemic heat tolerance across multiple traits. This added value could be significant since the economic selection indices, e.g. for the Australian dairy industry, are formulated to capture different aspects of farm profitability, including production, fertility, health, functional, and type as well as feed efficiency traits [39]. Selecting for thermotolerance would be advantageous if the goal is to simultaneously achieve an optimal level of heat tolerance for multiple traits [24]. Therefore, further studies are needed to investigate the benefits of sequence variants in improving heat tolerance with respect to other traits that are likely to be affected by heat and humidity, such as fertility and health traits.

## Conclusions

Our results show that the accuracy of genomic prediction for heat-tolerance milk yield traits (milk, fat, and protein) can be improved when the selected sequence variants linked to heat tolerance are added to the standard 50k SNP panel, with values ranging from 0.01 to 0.10 units depending on the prediction scenario. However, when predicting across breeds, adding informative sequence markers from the Holstein cow discovery set to the standard 50k SNP array (i.e., 50k + top SNPs from GWAS) decreased the accuracy of prediction in Jerseys compared to using only 50k SNP set, especially for the heat tolerance fat and protein yield traits. We observed improved predictions, particularly in the Jersey validation when using pre-selected markers from the multi-breed (Holstein + Jersey cows) SNP discovery set, where the reference population used included Holstein and Jersey bulls (i.e., the multi-breed reference set). Prioritised sequence markers from single-trait GWAS yielded greater accuracy than those from the multi-trait meta-analysis of slope traits. Overall, the results show that sequence variants can be prioritised to improve the accuracy of heat tolerance and has a direct application in the development of custom SNP arrays.

## Supplementary Information

**Additional file 1: Table S1.** Number (included in brackets), breed, and sex of animals with phenotypes and genotypes used for different study objectives. **Table S2.** Additive genetic variance (AG) and genomic heritability (${h}^{2}$) estimates for heat tolerance (slope) traits based on 50k SNP data for Holstein cows (N = 29,107), Holstein bulls (N = 3323), Jersey cows (N = 6338) and crossbred cows (N = 790). **Table S3.** Additive genetic variance (AG) and genomic heritability (${h}^{2}$) estimates for milk intercept traits based on 50k SNP data for Holstein cows (N = 29,107), Holstein bulls (N = 3323), Jersey cows (N = 6338) and crossbred cows (N = 790). **Table S4.** Number of informative markers defined as ‘top SNPs’ selected from single-trait GWAS and multi-trait meta-analyses of intercept traits in the Holstein discovery cow set (N = 20,623). **Table S5.** Number of SNPs with the same effect direction at different GWAS p-value cut-off and their corresponding false discovery rate (FDR) between Holstein bulls (N = 3323) versus Holstein cows (N = 1223) and Holstein bulls (N = 3323) versus Jersey cows (N = 6338). The false discovery rate effect direction (FDR ED) was computed following [39] and the conventional false discovery rate (FDR) was calculated following [47]. **Table S6.** Accuracy and the dispersion bias of predictions (in brackets) from non-linear Bayesian methods (BayesR and BayesRC) versus GBLUP models. **Table S7.** Number of overlapping selected ‘top SNPs’ between intercept and slope traits and the proportion of SNPs with the sample effect direction detected from the Holstein cow discovery set at a GWAS p-value cut-off of 0.001. **Table S8.** Correlation between estimated breeding values for heat tolerance (based on BayesR and BayesRC) versus Australian selection index and balanced performance index values for the Holstein validation cows (N = 1122) used in this study. **Table S9.** Correlation of GBV between whole (${GBVs}_{W}$) versus “partial” data (${GBVs}_{P}$), and the dispersion bias of predictions (${\mathrm{b}}_{\mathrm{w},\mathrm{ p}}$) in the Holstein validation cows (N = 1223) using a linear regression method [45] based on GBLUP and BayesR models.

**Additional file 2: Figure S1.** Manhattan plot of p values from single-trait GWAS results of heat tolerance milk (A), fat (B), protein (C) yield slope traits for the Holstein cow discovery set (N = 20,623). The dashed line represents p-value cut-off = 0.001. **Figure S2.** QQ-plot for heat tolerance milk (HTMYslope), fat (HTFYslope), and protein (HTPYslope) from GWAS of the Holstein cow discovery set (N = 20,623). **Figure S3.** Manhattan plot of p values from single-trait GWAS results of heat tolerance milk (A), fat (B), protein (C) yield slope traits for combined set of Holstein and Jersey cow discovery set (N = 25,766). The dashed line represents p-value cut-off = 0.001. **Figure S4.** QQ-plot for heat tolerance milk (HTMYslope), fat (HTFYslope), and protein (HTPYslope) from GWAS using a combined set of Holsteins + Jersey cows (N = 25,766). **Figure S5.** Minor allele frequency (MAF) distribution of the 50k SNP data and the selected ‘top SNPs’ (most significant) from the imputed-whole genome sequence variants. **Figure S6.** Accuracy of genomic predictions in Holsteins (A; N = 1223), Jersey (B; N = 6338), and Holstein–Jersey crossbreds (C; N = 790) validation cows for milk (MYint), fat (FYint) and protein (PYint) yield intercept traits from different SNP sets based on the BayesR and BayesRC methods: (a) standard 50k SNP array (50k; colored grey) (b) 50k + top SNPs selected from single-trait GWAS (colored blue) and multi-trait meta-analysis (colored orange) at a less stringent cut-off threshold of [− log10(p-value) ≥ 2] and a more stringent p-value of [− log10(p-value) ≥ 3]. The top SNPs were selected from GWAS of Holstein cows (N = 20,623). Vertical lines represent standard errors calculated from three (Holsteins) and two (Jersey) random validation subsets. **Figure S7.** Bias of genomic predictions in Holsteins (A; N = 1223), Jersey (B; N = 6338), and Holstein–Jersey crossbreds (C; N = 790) validation cows for milk (MYint), fat (FYint), and protein (PYint) yield intercept traits from different SNP sets based on the BayesR and BayesRC methods. **Figure S8.** Bias of genomic predictions in Holsteins (A; N = 1223), Jersey (B; N = 6338), and Holstein-Jersey crossbreds (C; N = 790) validation cows for heat tolerance milk (HTMYslope), fat (HTFYslope), and protein (HTPYslope) yield slope traits from different SNP sets based on the BayesR and BayesRC methods. **Figure S9.** QTL discovery using the single-breed (Holstein cows; left) and the across-breed (Holsteins + Jersey cows; right) discovery set.

## Data Availability

DataGene (DataGene Ltd., Melbourne, Australia; https://datagene.com.au/) are the custodians of the raw phenotype and genotype data of Australian dairy cows. Research-related requests for access to the data may be accommodated on a case-by-case basis.
