# Supplementary material for: Functionally prioritised whole-genome sequence variants improve the accuracy of genomic prediction for heat tolerance
Source: Genet Sel Evol. 2022 Feb 19;54:17. doi: 10.1186/s12711-022-00708-8 (PMC8858496; doi:10.1186/s12711-022-00708-8)
Supplement: Supplementary file 1 — Additional file 1: Table S1. Number (included in brackets), breed, and sex of animals with phenotypes and genotypes used for different study objectives. Table S2. Additive genetic variance (AG) and genomic heritability (${h}^{2}$h2) estimates for heat tolerance (slope) traits based on 50k SNP data for Holstein cows (N = 29,107), Holstein bulls (N = 3323), Jersey cows (N = 6338) and crossbred cows (N = 790). Table S3. Additive genetic variance (AG) and genomic heritability (${h}^{2}$h2) estimates for milk intercept traits based on 50k SNP data for Holstein cows (N = 29,107), Holstein bulls (N = 3323), Jersey cows (N = 6338) and crossbred cows (N = 790). Table S4. Number of informative markers defined as ‘top SNPs’ selected from single-trait GWAS and multi-trait meta-analyses of intercept traits in the Holstein discovery cow set (N = 20,623). Table S5. Number of SNPs with the same effect direction at different GWAS p-value cut-off and their corresponding false discovery rate (FDR) between Holstein bulls (N = 3323) versus Holstein cows (N = 1223) and Holstein bulls (N = 3323) versus Jersey cows (N = 6338). The false discovery rate effect direction (FDR ED) was computed following [39] and the conventional false discovery rate (FDR) was calculated following [47]. Table S6. Accuracy and the dispersion bias of predictions (in brackets) from non-linear Bayesian methods (BayesR and BayesRC) versus GBLUP models. Table S7. Number of overlapping selected ‘top SNPs’ between intercept and slope traits [file 12711_2022_708_MOESM1_ESM.docx]

# Additional tables for:

# Functionally prioritised whole-genome sequence variants improve the accuracy of genomic prediction for heat tolerance

**Evans K. Cheruiyot^1,2^, Mekonnen Haile-Mariam^2*^, Benjamin G. Cocks^1,2^, Iona M. MacLeod^2^, Raphael Mrode^3,4^, Jennie E. Pryce^1,2^**

^1^School of Applied Systems Biology, La Trobe University, Bundoora, Victoria 3083, Australia

^2^Agriculture Victoria Research, AgriBio, Centre for AgriBiosciences, Bundoora, Victoria 3083, Australia

^3^International Livestock Research Institute, Nairobi, Kenya

^4^Scotland’s Rural College, Edinburgh, United Kingdom

**Table S1** Number (included in brackets), breed, and sex of animals with phenotypes and genotypes used for different study objectives.

|  | QTL discovery and Reference set | | Validation sets | | |
| --- | --- | --- | --- | --- | --- |
|  | QTL discovery | Reference set | Holsteins ♀ | Jersey ♀* | Crossbreds ♀ |
| Scenario 1 | Holsteins (20,623 ♀) | Holsteins (3323 ♂) | Subset 1 (600) Subset 2 (623) | Subset 1 (3169) Subset 2 (3169) | Subset 1 (395)  Subset 2 (395) |
| Scenario 2 | Holstein (20,623 ♀) + Jersey (5,143 ♀) | Holsteins (3323 ♂) | Subset 1 (600) Subset 2 (623) | Subset 1 (590) Subset 2 (605) | Subset 1 (395)  Subset 2 (395) |
| Scenario 3 | Holsteins (20,623 ♀) | Holsteins (3323 ♂) +  Jersey (852 ♂) | Subset 1 (600) Subset 2 (623) | Subset 1 (200)  Subset 2 (231) | Subset 1 (395)  Subset 2 (395) |

Scenario 1 – aimed at testing the added value of selected sequence variants from single-breed (Holsteins) in the genomic prediction of heat tolerance; Scenario 2 – using a multi-breed (Holsten + Jersey) population to select informative markers for heat tolerance; Scenario 3 – same as ‘scenario 1’ but using multi-breed (Holstein + Jersey) reference set instead of the single-breed population (Holsteins); *the number of validation animals that remained after splitting cows into a) discovery set (‘scenario 2’) and reference set (‘scenario 3’).

**Table S2** Additive genetic variance (AG) and genomic heritability ($h^{2}$) estimates for heat tolerance (slope) traits based on 50k SNP data for Holstein cows (N = 29,107), Holstein bulls (N = 3323), Jersey cows (N = 6338) and crossbred cows (N = 790).

| Breed/population |  | Heat tolerance slope traits | |
| --- | --- | --- | --- |
| Holstein cows | Trait | AG ± SE | $h^{2}$ ± SE |
|  | HTMYslope | 4.80 (0.20) | 0.23 (0.01) |
|  | HTFYslope | 0.56 (0.02) | 0.21 (0.01) |
|  | HTPYslope | 0.47 (0.02) | 0.20 (0.01) |
| Holstein bulls | HTMYslope | 2.16 (0.25) | 0.27 (0.03) |
|  | HTFYslope | 0.24 (0.03) | 0.26 (0.03) |
|  | HTPYslope | 0.23 (0.03) | 0.25 (0.03) |
| Jersey cows | HTMYslope | 3.0 (0.25) | 0.26 (0.02) |
|  | HTFYslope | 0.41 (0.04) | 0.23 (0.03) |
|  | HTPYslope | 0.44 (0.04) | 0.25 (0.02) |
| Crossbred cows | HTMYslope | 12.11 (2.44) | 0.58 (0.10) |
|  | HTFYslope | 1.0 (0.33) | 0.34 (0.11) |
|  | HTPYslope | 1.63 (0.37) | 0.51 (0.10) |

**Table S3** Additive genetic variance (AG) and genomic heritability ($h^{2}$) estimates for milk intercept traits based on 50k SNP data for Holstein cows (N = 29,107), Holstein bulls (N = 3323), Jersey cows (N = 6338) and crossbred cows (N = 790).

| Breed/population |  | Intercept traits | |
| --- | --- | --- | --- |
| Holstein cows | Trait | AG ± SE | $h^{2}$ ± SE |
|  | MYint | 381.00 (14.35) | 0.36 (0.01) |
|  | FYint | 38.79 (1.64) | 0.30 (0.01) |
|  | PYint | 0.18 (0.89) | 0.24 (0.01) |
| Holstein bulls | MYint | 219.72 (20.31) | 0.40 (0.03) |
|  | FYint | 16.17 (1.83) | 0.30 (0.03) |
|  | PYint | 11.38 (1.22) | 0.32 (0.03) |
| Jersey cows | MYint | 256.46 (15.57) | 0.43 (0.02) |
|  | FYint | 29.70 (2.32) | 0.30 (0.02) |
|  | PYint | 15.29 (1.22) | 0.28 (0.02) |
| Crossbred cows | MYint | 772 (144.10) | 0.64 (0.10) |
|  | FYint | 39.96 (13.66) | 0.32 (0.10) |
|  | PYint | 36.09 (8.51) | 0.48 (0.10) |

**Table S4** Number of informative markers defined as ‘top SNPs’ selected from single-trait GWAS and multi-trait meta-analyses of intercept traits in Holstein discovery cow set (N = 20,623).

| Trait | Top SNPs (logP = 2) | Top SNPs (logP = 3) |
| --- | --- | --- |
| MYint | 9587 (52,159) | 1917 (44,489) |
| FYint | 9805 (52,377) | 1780 (44,352) |
| PYint | 10,132 (52,704) | 1865 (44,437) |
| Meta-GWAS | 9041 (51,613) | 2634 (45,206) |

Markers were selected based on the GWAS cut-off thresholds of -log10(p-value) ≥ 2 and -log10(p-value) ≥ 3; The values in brackets are the final number of SNPs after adding selected ‘top SNPs’ to the 50k SNP data used in the BayesRC analyses (i.e., 42,572 SNPs + top SNPs); Traits are defined as milk (MYint), fat (FYint) and protein (PYint) yield intercept traits.

**Table S5** Number of SNPs with the same effect direction at different GWAS P-value cut-off and their corresponding false discovery rate (FDR) between Holstein bulls (N = 3323) versus Holstein cows (N = 1223) and Holstein bulls (N = 3323) versus Jersey cows (N = 6338).

|  | Holstein ♂ versus Holstein ♀ | | | | | Holstein ♂ versus Jersey ♀ | | | | |
| --- | --- | --- | --- | --- | --- | --- | --- | --- | --- | --- |
| Trait | P-value^1^  cut-off | N Sig.^2^ | N Same^3^ | FDR ED^4^ | FDR^5^ | P-value  cut-off | N Sig. | N Same | FDR ED | FDR |
| HTMYslope | 0.1 | 170,742 | 115,086 | 0.651931 | 1 | 0.1 | 117,488 | 58,342 | 1 | 1 |
| HTMYslope | 0.01 | 3734 | 2871 | 0.462239 | 1 | 0.01 | 3779 | 2462 | 0.69701 | 1 |
| **HTMYslope** | **0.001** | **493** | **420** | **0.296146** | **1** | **0.001** | **1080** | **774** | **0.566667** | **1** |
| HTMYslope | 1.00E-04 | 134 | 111 | 0.343284 | 1 | 1.00E-04 | 667 | 525 | 0.425787 | 1 |
| HTMYslope | 1.00E-05 | 6 | 6 | 0 | 1 | 1.00E-05 | 525 | 471 | 0.205714 | 0.212229 |
| HTMYslope | 1.00E-06 | 0 | 0 | NA | NA | 1.00E-06 | 395 | 349 | 0.232911 | 0.028208 |
| HTMYslope | 1.00E-07 | 0 | 0 | NA | NA | 1.00E-07 | 227 | 183 | 0.387665 | 0.004908 |
| HTMYslope | 1.00E-08 | 0 | 0 | NA | NA | 1.00E-08 | 220 | 177 | 0.390909 | 0.000506 |
| HTMYslope | 1.00E-09 | 0 | 0 | NA | NA | 1.00E-09 | 199 | 158 | 0.41206 | 5.60E-05 |
| HTMYslope | 1.00E-10 | 0 | 0 | NA | NA | 1.00E-10 | 108 | 68 | 0.740741 | 1.03E-05 |
|  |  |  |  |  |  |  |  |  |  |  |
| HTFYslope | 0.1 | 157,766 | 98,963 | 0.745446 | 1 | 0.1 | 118,672 | 59,655 | 0.994624 | 1 |
| HTFYslope | 0.01 | 4104 | 3594 | 0.248538 | 1 | 0.01 | 2012 | 1203 | 0.804175 | 1 |
| **HTFYslope** | **0.001** | **1304** | **1240** | **0.09816** | **1** | **0.001** | **738** | **524** | **0.579946** | **1** |
| HTFYslope | 1.00E-04 | 888 | 829 | 0.132883 | 1 | 1.00E-04 | 299 | 172 | 0.849498 | 1 |
| HTFYslope | 1.00E-05 | 645 | 618 | 0.083721 | 0.219504 | 1.00E-05 | 182 | 88 | 1 | 0.612217 |
| HTFYslope | 1.00E-06 | 547 | 547 | 0 | 0.025883 | 1.00E-06 | 162 | 78 | 1 | 0.068779 |
| HTFYslope | 1.00E-07 | 509 | 509 | 0 | 0.002782 | 1.00E-07 | 149 | 77 | 0.966443 | 0.007478 |
| HTFYslope | 1.00E-08 | 253 | 253 | 0 | 0.00056 | 1.00E-08 | 121 | 77 | 0.727273 | 0.000921 |
| HTFYslope | 1.00E-09 | 2 | 2 | 0 | 0.007079 | 1.00E-09 | 118 | 77 | 0.694915 | 9.44E-05 |
| HTFYslope | 1.00E-10 | 1 | 1 | 0 | 0.001416 | 1.00E-10 | 117 | 77 | 0.683761 | 9.52E-06 |
|  |  |  |  |  |  |  |  |  |  |  |
| HTPYslope | 0.1 | 150,750 | 57,437 | 1 | 1 | 0.1 | 114,636 | 56,009 | 1 | 1 |
| HTPYslope | 0.01 | 3050 | 1211 | 1 | 1 | 0.01 | 1818 | 629 | 1 | 1 |
| **HTPYslope** | **0.001** | **1** | **0** | **1** | **1** | **0.001** | **4** | **2** | **1** | **1** |
| HTPYslope | 1.00E-04 | 0 | 0 | NA | NA | 1.00E-04 | 0 | 0 | NA | NA |
| HTPYslope | 1.00E-05 | 0 | 0 | NA | NA | 1.00E-05 | 0 | 0 | NA | NA |
| HTPYslope | 1.00E-06 | 0 | 0 | NA | NA | 1.00E-06 | 0 | 0 | NA | NA |
| HTPYslope | 1.00E-07 | 0 | 0 | NA | NA | 1.00E-07 | 0 | 0 | NA | NA |
| HTPYslope | 1.00E-08 | 0 | 0 | NA | NA | 1.00E-08 | 0 | 0 | NA | NA |
| HTPYslope | 1.00E-09 | 0 | 0 | NA | NA | 1.00E-09 | 0 | 0 | NA | NA |
| HTPYslope | 1.00E-10 | 0 | 0 | NA | NA | 1.00E-10 | 0 | 0 | NA | NA |

^1^GWAS cut-off p-value imposed on both populations; ^2^The number of significant SNPs at this cut-off p-value; ^3^the number of significant SNPs with the same effect direction in both population; ^4^false discovery rate effect direction (FDR ED) computed following [39]; ^5^conventional false discovery rate (FDR) calculated following [47]; HTMYslope, HTFYslope, and HTPYslope represents heat tolerance milk, fat, and protein yield slope traits, respectively.

**Table S6** Accuracy and the dispersion bias of predictions (in brackets) from non-linear Bayesian methods (BayesR and BayesRC) versus GBLUP models.

|  | Bayesian models | | GBLUP models | | |
| --- | --- | --- | --- | --- | --- |
| Trait | 50k SNPs (BayesR) | 50k + top SNPs (BayesRC) | 50k SNPs | 50k + top SNPs (1 GRM) | 50k + top SNPs (2 GRMs) |
| HTMYslope | 0.49 (1.44) | 0.49 (1.33) | 0.44 (1.58) | 0.47 (1.57) | 0.46 (1.38) |
| HTFYslope | 0.49 (1.32) | 0.53 (1.32) | 0.36 (1.33) | 0.44 (1.47) | 0.49 (1.44) |
| HTPYslope | 0.39 (1.10) | 0.45 (1.21) | 0.42 (1.35) | 0.44 (1.37) | 0.45 (1.30) |

GRM – genomic relationship matrix; top SNPs were pre-selected from GWAS of 20,623 Holstein cows; HTMYslope, HTMYslope and HTMYslope represents heat tolerance milk, fat, protein yield slope traits, repetitively.

**Table S7** Number of overlapping selected ‘top SNPs’ between intercept and slope traits and the proportion of SNPs with the sample effect direction detected from Holstein cow discovery set at GWAS p-value cut-off of 0.001.

|  | N sig. Intercept | N sig. Slope | Overlap | Same effect dir. |
| --- | --- | --- | --- | --- |
| HT Milk yield | 9318 | 9207 | 991 | 100% |
| HT Fat yield | 9500 | 9352 | 1578 | 0.0% |
| HT Protein yield | 9876 | 9633 | 2017 | 0.0% |

HT – heat tolerance

**Table S8** Correlation between estimated breeding values for heat tolerance (based on BayesR and BayesRC) versus Australian selection index and balanced performance index values for the Holstein validation cows (N = 1122) used in this study.

|  | Industry-standard 50k | | 50k + top SNPs (SS-GWAS) | | 50k + top SNPs (Meta-GWAS) | |
| --- | --- | --- | --- | --- | --- | --- |
| Trait | ASI | BPI | ASI | BPI | ASI | BPI |
| HTMYslope | -0.02 | 0.06 | -0.02 | 0.08 | -0.03 | 0.10 |
| HTFYslope | -0.10 | -0.15 | -0.07 | -0.15 | -0.08 | -0.16 |
| HTMYslope | -0.07 | -0.07 | -0.06 | -0.09 | -0.07 | -0.09 |

ASI – Australian Selection Index (used for ranking most profitable daughters based on milk, fat, and protein yield); BPI – Australian Balanced performance index; HTMYslope, HTFYslope, HTPYslope represents heat tolerance milk, fat, and protein yield slope traits; the ‘top SNPs’ were preselected from either single-trait GWAS (SS-GWAS) or meta-analysis of SS-GWAS results of slope traits (Meta-GWAS) of Holstein discovery set (N = 20,623) based on cut-off p-value of 0.001.

**Table S9** Correlation of GBVs between whole (${GBVs}_{W}$) versus “partial” data (${GBVs}_{P}$), and the dispersion bias of predictions ($b_{w, p}$) in the Holstein validation cows (N = 1223) using linear regression method [44] based on GBLUP and BayesR models.

|  | GBLUP | | BayesR | |
| --- | --- | --- | --- | --- |
|  | ${r(u}_{p},u_{w})$± SE | $b_{w, p}$ ± SE | ${r(u}_{p},u_{w})$± SE | $b_{w, p}$ ± SE |
| HTMYslope | 0.85 ± 0.01 | 1.12 ± 0.02 | 0.89 ± 0.01 | 0.99 ± 0.01 |
| HTFYslope | 0.86 ± 0.01 | 0.94 ± 0.02 | 0.91 ± 0.01 | 1.05 ± 0.01 |
| HTPYslope | 0.89 ± 0.01 | 1.03 ± 0.01 | 0.89 ± 0.01 | 0.93 ± 0.01 |

*Partial data were generated by excluding 50% of the reference Holstein bulls from the analysis.
