# Supplementary material for: Functionally prioritised whole-genome sequence variants improve the accuracy of genomic prediction for heat tolerance
Source: Genet Sel Evol. 2022 Feb 19;54:17. doi: 10.1186/s12711-022-00708-8 (PMC8858496; doi:10.1186/s12711-022-00708-8)
Supplement: Supplementary file 2 — Additional file 2: Figure S1. Manhattan plot of p values from single-trait GWAS results of heat tolerance milk (A), fat (B), protein (C) yield slope traits for the Holstein cow discovery set (N = 20,623). The dashed line represents p-value cut-off = 0.001. Figure S2. QQ-plot for heat tolerance milk (HTMYslope), fat (HTFYslope), and protein (HTPYslope) from GWAS of the Holstein cow discovery set (N = 20,623). Figure S3. Manhattan plot of p values from single-trait GWAS results of heat tolerance milk (A), fat (B), protein (C) yield slope traits for combined set of Holstein and Jersey cow discovery set (N = 25,766). The dashed line represents p-value cut-off = 0.001. Figure S4. QQ-plot for heat tolerance milk (HTMYslope), fat (HTFYslope), and protein (HTPYslope) from GWAS using a combined set of Holsteins + Jersey cows (N = 25,766). Figure S5. Minor allele frequency (MAF) distribution of the 50k SNP data and the selected ‘top SNPs’ (most significant) from the imputed-whole genome sequence variants. Figure S6. Accuracy of genomic predictions in Holsteins (A; N = 1223), Jersey (B; N = 6338), and Holstein–Jersey crossbreds (C; N = 790) validation cows for milk (MYint), fat (FYint) and protein (PYint) yield intercept traits from different SNP sets based on the BayesR and BayesRC methods: (a) standard 50k SNP array (50k; colored grey) (b) 50k + top SNPs selected from single-trait GWAS (colored blue) and multi-trait meta-analysis (colored orange) at a less stringent cut-off threshold of [− log10(p-value) ≥ 2] and a more stringent p-value of [− log10(p-value) ≥ 3]. The top SNPs were selected from GWAS of Holstein cows (N = 20,623). Vertical lines represent standard errors calculated from three (Holsteins) and two (Jersey) random validation subsets. Figure S7. Bias of genomic predictions in Holsteins (A; N = 1223), Jersey (B; N = 6338), and Holstein–Jersey crossbreds (C; N = 790) validation cows for milk (MYint), fat (FYint), and protein (PYint) yield intercept traits from dif [file 12711_2022_708_MOESM2_ESM.docx]

# Additional figures for:

# Functionally prioritised whole-genome sequence variants improve the accuracy of genomic prediction for heat tolerance

**Evans K. Cheruiyot^1,2^, Mekonnen Haile-Mariam^2*^, Benjamin G. Cocks^1,2^, Iona M. MacLeod^2^, Raphael Mrode^3,4^, Jennie E. Pryce^1,2^**

^1^School of Applied Systems Biology, La Trobe University, Bundoora, Victoria 3083, Australia

^2^Agriculture Victoria Research, AgriBio, Centre for AgriBiosciences, Bundoora, Victoria 3083, Australia

^3^International Livestock Research Institute, Nairobi, Kenya

^4^Scotland’s Rural College, Edinburgh, United Kingdom










**Figure S1** Manhattan plot of p values from single-trait GWAS results of heat tolerance milk (A), fat (B), protein (C) yield slope traits for Holstein cow discovery set (N = 20,623). The dashed line represents p-value cut-off = 0.001.


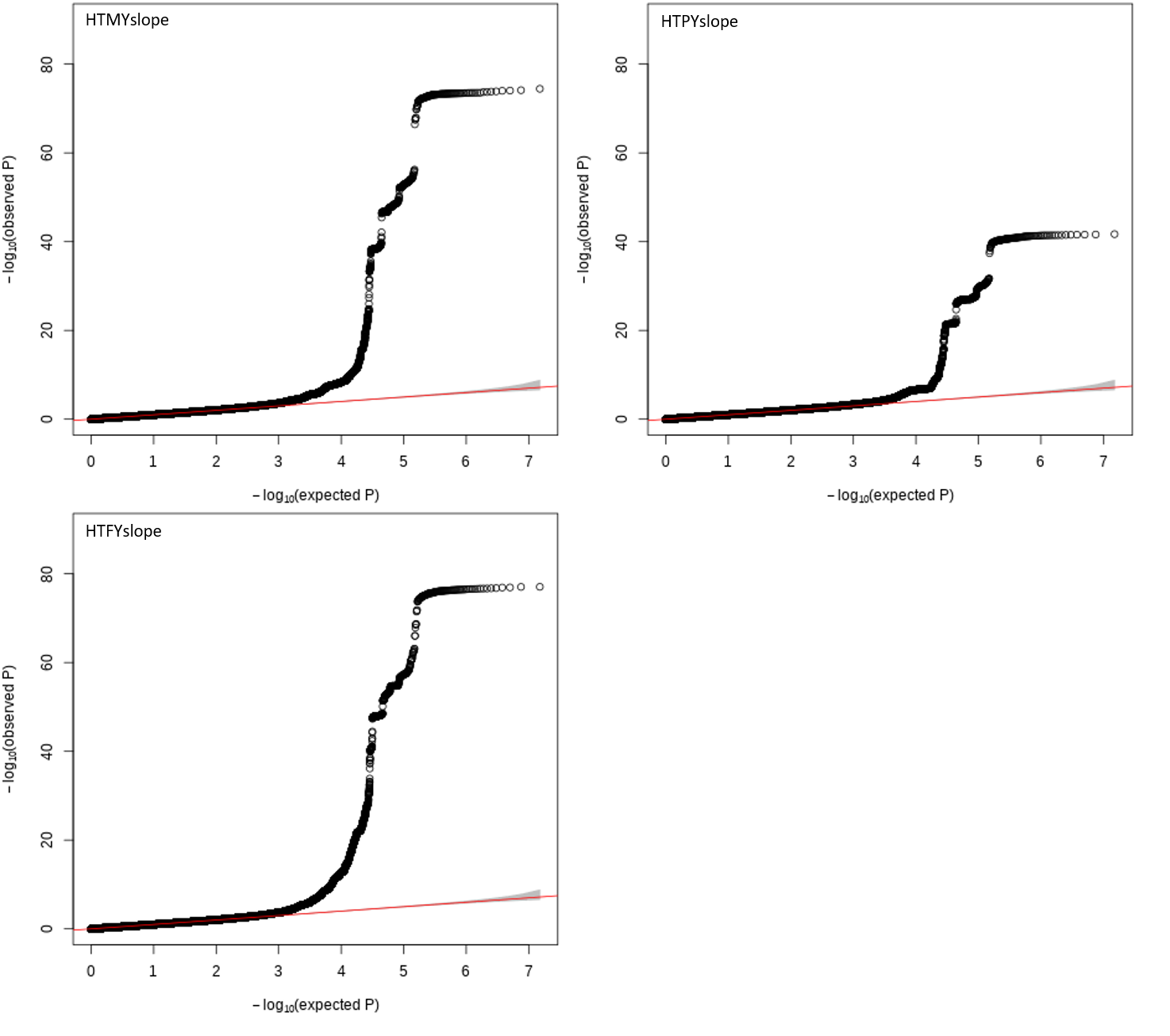


**Figure S2** QQ-plot for heat tolerance milk (HTMYslope), fat (HTFYslope), and protein (HTPYslope) from GWAS of Holstein cow discovery set (N = 20,623).


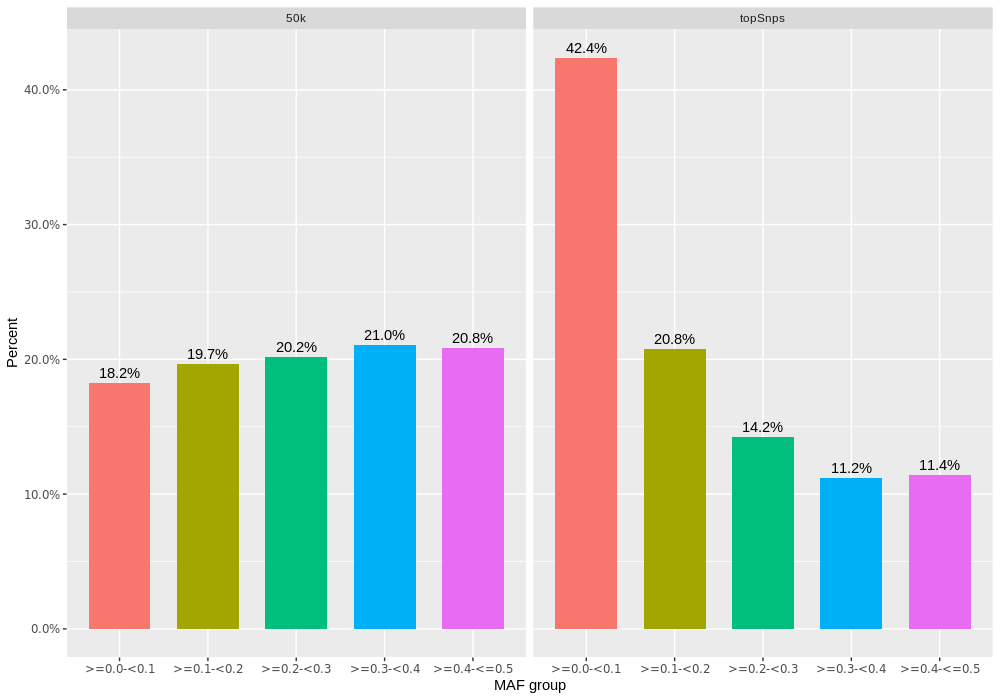


**Figure S3** Minor allele frequency (MAF) distribution of the 50k SNP data and the selected ‘top SNPs’ (most significant) from the imputed-whole genome sequence variants.











**Figure S4** Manhattan plot of p values from single-trait GWAS results of heat tolerance milk (A), fat (B), protein (C) yield slope traits for combined set of Holstein and Jersey cow discovery set (N = 25,766). The dashed line represents p-value cut-off = 0.001.


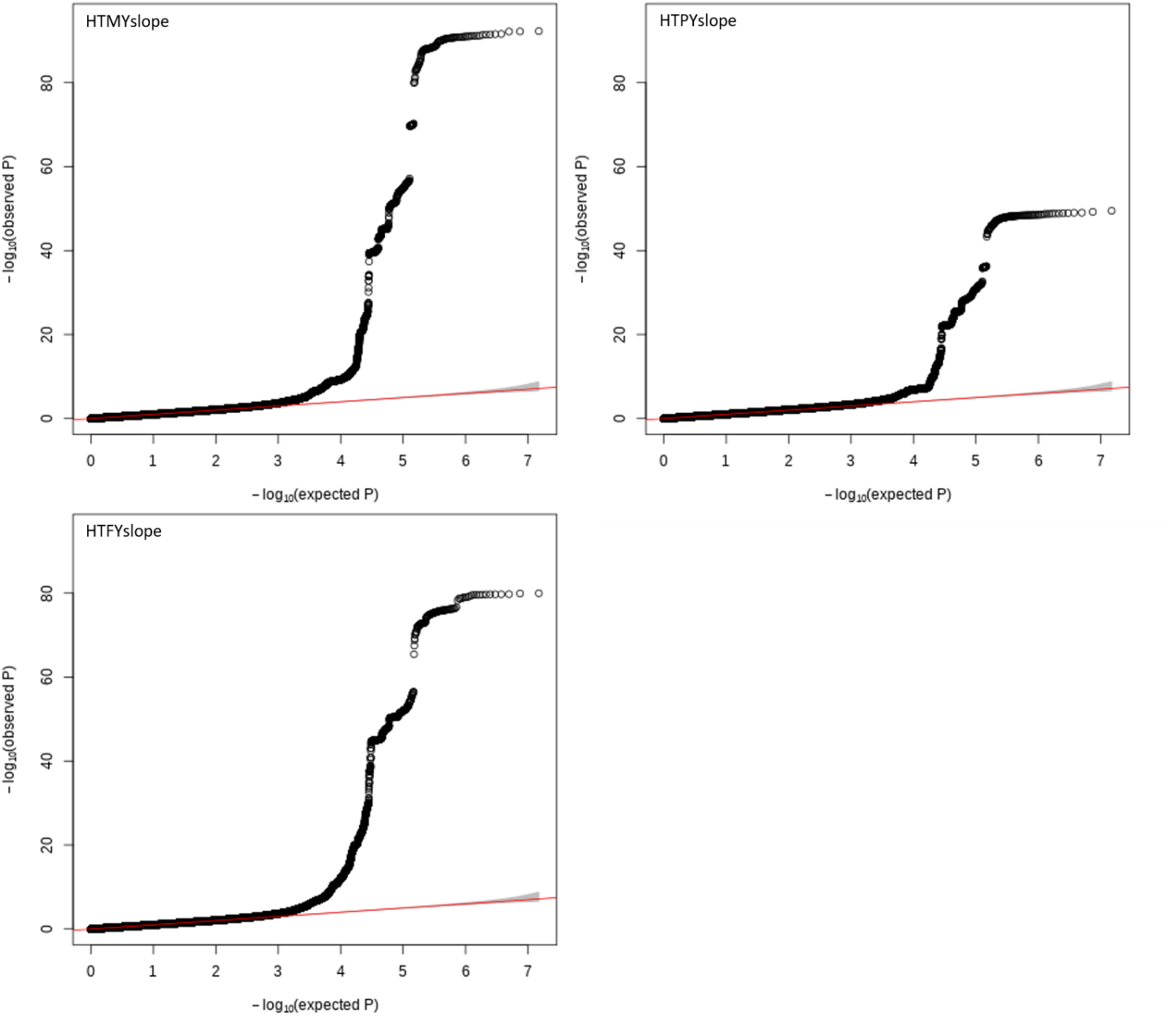


**Figure S5** QQ-plot for heat tolerance milk (HTMYslope), fat (HTFYslope), and protein (HTPYslope) from GWAS using a combined set of Holsteins + Jersey cows (N = 25,766).


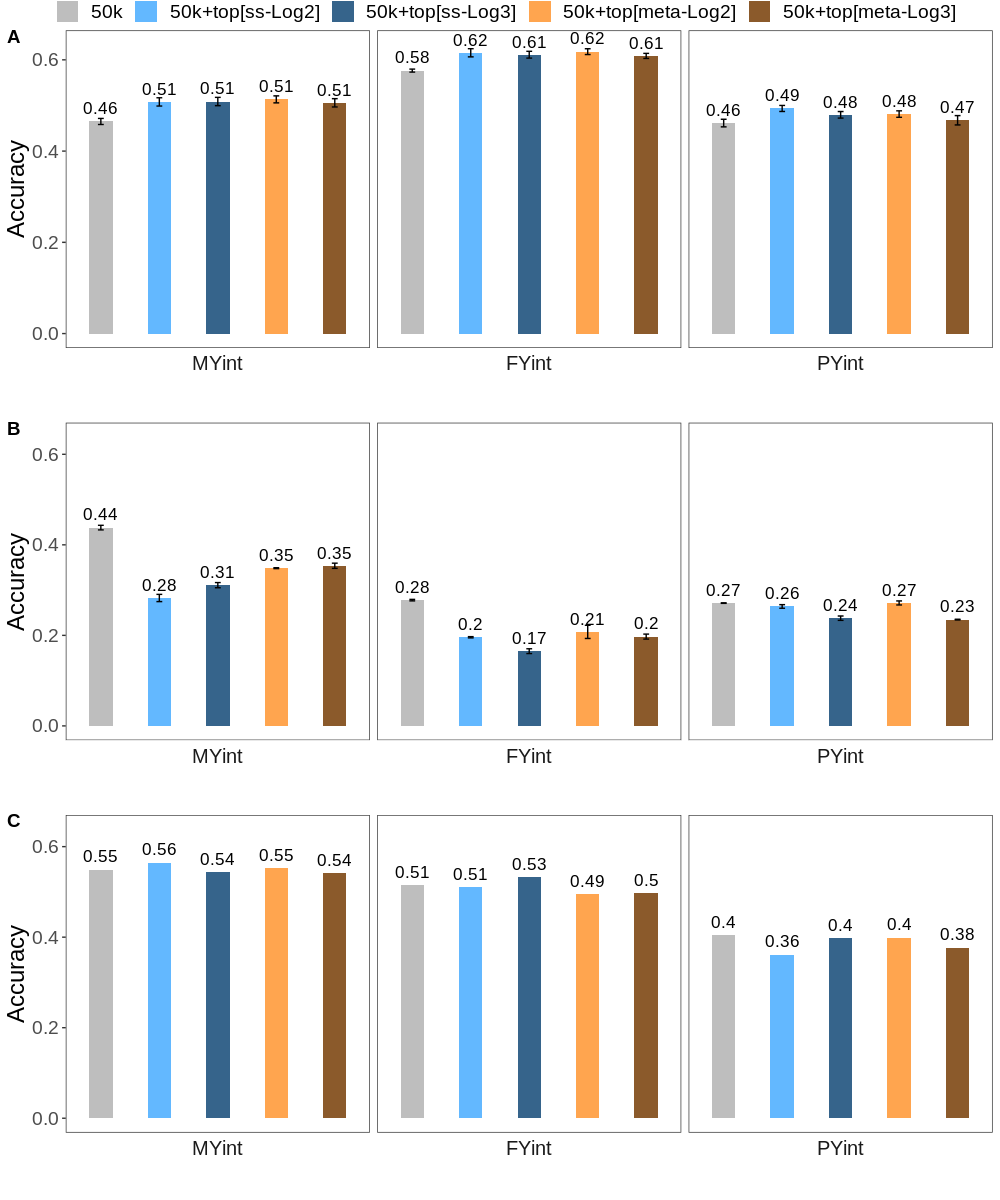


**Figure S6** Accuracy of genomic predictions in Holsteins (**A;** N=1223), Jersey (**B;** N=6338), and Holstein-Jersey crossbreds (**C;** N=790) validation cows for milk (MYint), fat (FYint) and protein (PYint) yield intercept traits from different SNP sets based on the BayesR and BayesRC method: a) standard 50k SNP array (50k; colored grey) b) 50k + top SNPs selected from single-trait GWAS (colored blue) and multi-trait meta-analysis (colored orange) at less stringent cut-off threshold of [-log10(p-value) ≥ 2] and more stringent p-value of [-log10(p-value) ≥ 3]. The top SNPs were selected from GWAS of Holstein cows (N = 20,623). Vertical lines represent standard errors calculated from three (Holsteins) and two (Jersey) random validation subsets.


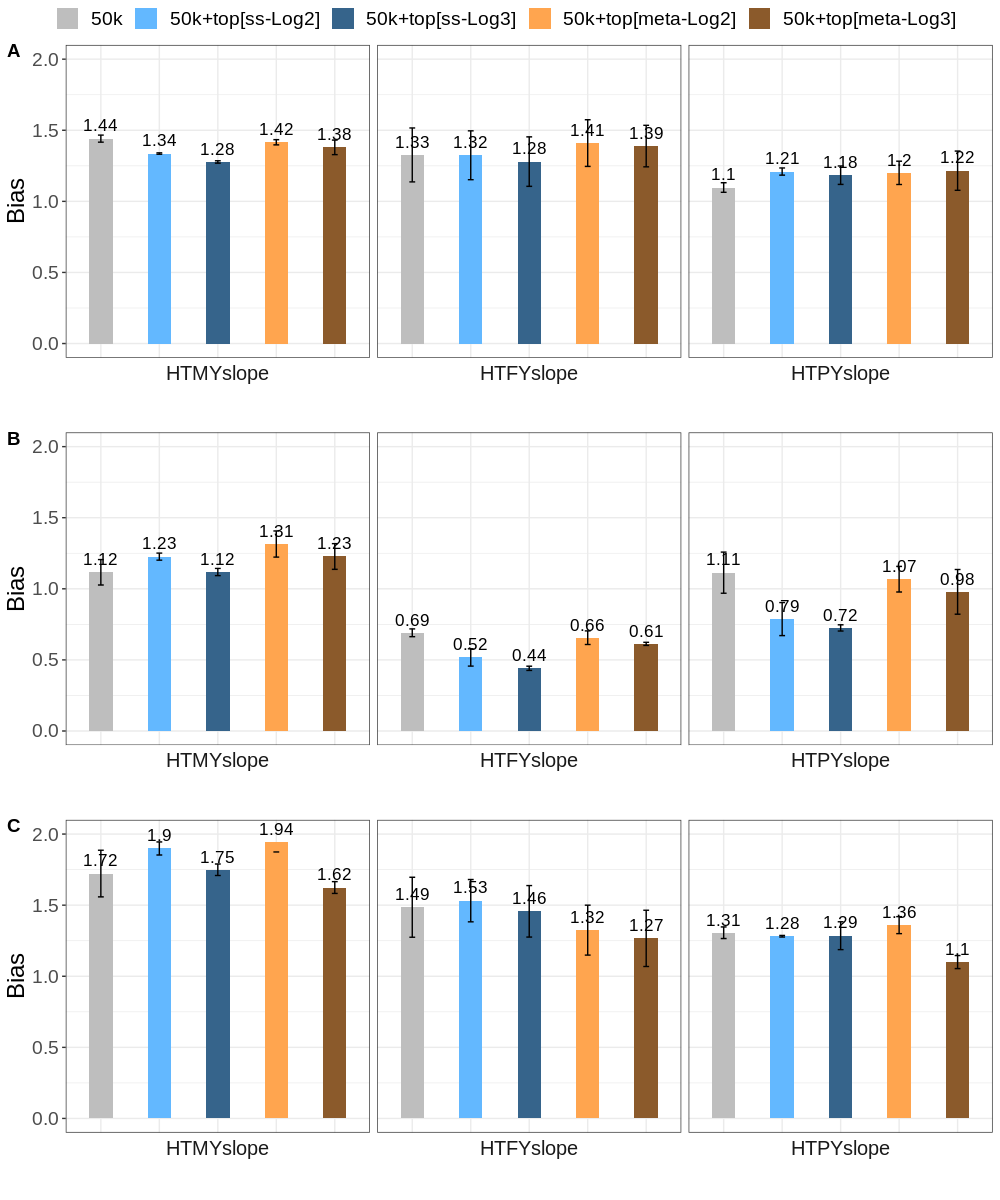


**Figure S7** Bias of genomic predictions in Holsteins (**A;** N = 1223), Jersey (**B;** N = 6338), and Holstein-Jersey crossbreds (**C;** N = 790) validation cows for heat tolerance milk (HTMYslope), fat (HTFYslope), and protein (HTPYslope) yield slope traits from different SNP sets based on the BayesR and BayesRC methods.


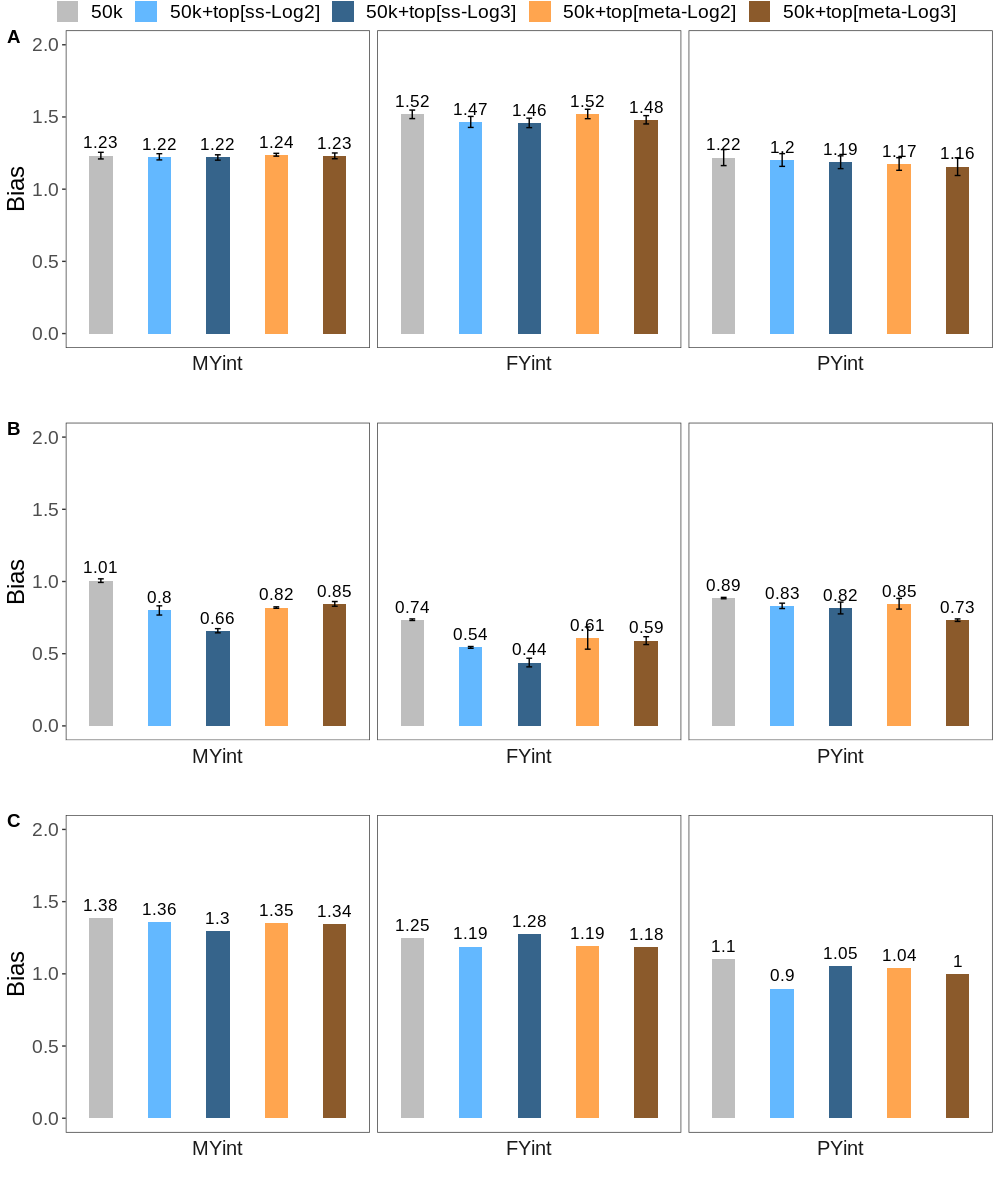


**Figure S8** Bias of genomic predictions in Holsteins (**A;** N = 1223), Jersey (**B;** N = 6338), and Holstein-Jersey crossbreds (**C;** N = 790) validation cows for milk (MYint), fat (FYint), and protein (PYint) yield intercept traits from different SNP sets based on the BayesR and BayesRC method.


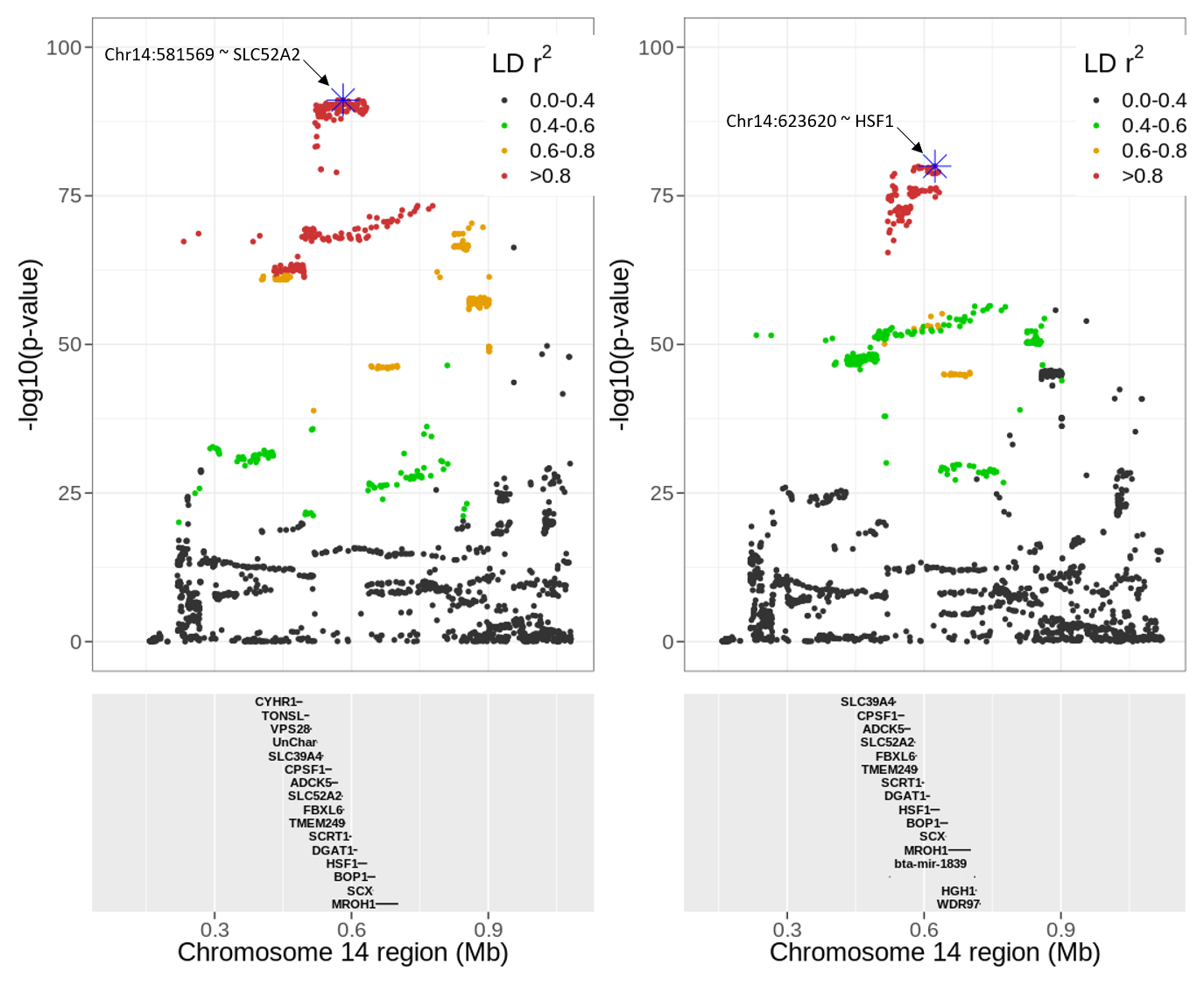


**Figure S9** QTL discovery using single-breed (Holstein cows; left) and across-breed (Holsteins + Jersey cows; right) discovery set.
